# Supplementary material for: Chronic platelet-derived growth factor receptor signaling exerts control over initiation of protein translation in glioma
Source: Life Sci Alliance. 2018 Jun 19;1(3):e201800029. doi: 10.26508/lsa.201800029 (PMC6238596; doi:10.26508/lsa.201800029)
Supplement: Supplementary file 1 [file LSA-2018-00029_Preparation_of_compounds.zip › LSA-2018-00029_Supplementary_Materials_and_Methods.pdf]

Supplementary Materials and Methods

Curation criteria for the PDGFR signaling map

The information used to build the PDGFR pathway map was curated manually by ourselves. We used a top down approach, initiating the map using PDGFR related keywords, and performing several iterative searches with new keywords through the following steps.

Step 1: We performed keyword searches on PUBMED for publications related to: PDGFR, PDGFRA, PDGFRB, PDGFRα, PDGFRβ, PDGF-A, PDGF-B, PDGF-C, PDGF-D, kinase activity, phosphorylation, signaling, signal transduction. We screened titles and abstracts and a decision is made to include or exclude the manuscripts for further assessment based on whether the abstract contained relevant information on PDGFR and downstream signaling.

Step 2: Manuscripts that have made it through the first stage are obtained and analyzed to make a more detailed assessment. References were selected based on the following inclusion and exclusion criteria:

1) Published paper are written in English.
2) Studies on PDGFR pathway and related signaling reactions in any species and disease models.
3) For each reaction, the earliest original study is included. Important review articles can be also included for complex molecular interactions.
4) Publications with low quality or experimental details or unclear conclusions are not included.

Step 3: Once the map was initiated, second and third rounds of keyword searches were performed using terms (for example): ras signaling, plcgamma singaling, mTORC1/2 signaling etc. Process to Step 2 was repeated.

Step 4: The accuracy of curation materials was confirmed by an independent curator.

Step 5: Our preliminary map was cross-referenced to EGFR and mTOR maps (Oda et al., Mol Syst Biol, 2005, Caron et al., Mol Syst Biol, 2010) for accuracy and consistency.

Conditional PDGFRα transgenic strain

The PDGFRα genetically engineered mouse strain was constructed as follows: wild-type human PDGFRα cDNA (obtained from Dr. Andrius Kazlauskas) was inserted in the CAGGS-Col1α1 targeting vector plasmid (obtained from R. Jaenisch, Whitehead Institute, Cambridge, MA) and was co-electroporated with pCAGGS-Flpe plasmid into C2 ES cells (R. Jaenisch, Whitehead Institute, Cambridge, MA). Following clonal hygromycin selection, individual clones were screened by Southern blot hybridization with probes described elsewhere (Beard C, *et al*. Efficient method to generate single- copy transgenic mice by site-specific integration in embryonic stem cells. Genesis 44, 23-28 (2006)). Knocked-in PDGFRα transgene ES cloneswere used to produce chimeric mice, which were then mated to generate founder animals. Germline-transmitted LSL-hPDGFRα founder males were mated to conditional Tp53 mice (Marino S, *et al*. Induction of medulloblastomas in p53-null mutant mice by somatic inactivation of Rb in the external granular layer cells of the cerebellum. *Genes Dev* **14**, 994-1004 (2000)). Compound LSL-hPDGFRα;p53$^{lox/lox}$ transgenic mice displayed no abnormalities and are healthy (data not shown). PCR genotype for the LSL-hPDGFRα transgenic mice using genomic DNA isolated from tail biopsies is accomplished using the following primer set: Col frt A1 (5'GCA CAG CAT TGC GGA CAT GC3'), Col frt B (5'CCC TCC ATG TGT GAC CAA GG3'), and Col frt C (5'GCA GAA GCG CGG CCG TCT GG3') for the collagen1α1 locus genotype. The PCR cycling parameters are 94°C 5 min, 35 cycles at 94°C for 30 sec, 55°C for 30 sec, and 72°C for 30 sec followed by a 10-min extension at 72°C. Genotyping protocol for the Tp53 conditional strain is described elsewhere (Marino et al, 2000).

Virus construct, production and determination of titer

We modified the pSLIK lentivector system (Shin KJ*, et al.* A single lentiviral vector platform for microRNA-based conditional RNA interference and coordinated transgene expression. *Proc Natl Acad Sci U S A* **103**, 13759- 13764 (2006)) to express the human PDGF-A cDNA and Cre recombinase. Viruses are produced by cotransfection of 293T cells with packaging vectors and purified by ultracentrifugation of conditioned media, resuspended in PBS, aliquoted and stored at -80°C. Standardization for intracranial injections with identical viral titers was achieved by functiona tittering of viral preparations for Cre activity by serial dilution infection of immortalized ear fibroblasts derived from Cdkn2a-null conditional LSL-tdTomato mice (Ai9 reporter strain) (Madisen L*, et al.* A robust and high-throughput Cre reporting and characterization system for the whole mouse brain. *Nat Neurosci* **13**, 133-140 (2010)).

Intracranial stereotactic injections

Adult animals (>=3 months of age) of the indicated genotype were anesthetized with an IP injection of ketamine/xylazine (ketamine 100 -125 mg/kg, xylazine 10–12.5 mg/kg). The animals were mounted in a Stoelting stereotaxic frame (Harvard Apparatus Inc.) with nonpuncturing ear bars. The incision site was shaved and sterilized with betadine surgical scrub, and a single incision was made from the anterior pole of the skull to the posterior ridge. A 1-mm burr hole was drilled at the stereotactically defined location of the striatum (2.1 mm rostral to the bregma, 1.5 mm lateral to the midline, and at 2 mm depth to the pia surface) and either a 1µl Hamilton syringe or a pulled glass pipette mounted onto a Nanoject II injector (Drummond Scientific Company) was used to inject the lenti-PDGFA-Cre virus at a rate of 0.1 uL/min. Following retraction of the syringe or pipette, the burr hole was filled with sterile bone wax, the skin drawn up and sutured, and the animal placed in a cage with a padded bottom atop a surgical heat pad until ambulatory.

Statistical Analysis

Statistical analyses were carried out using GraphPad Prism 7. Two-tailed Student’s t tests were used for single comparison. Significance for survival analyses was determined by the log rank (Mantel- Cox) test. p values of less than 0.05 were considered statistically significant. The Kolmogorov–Smirnov tests (KS test) for different functional groups and kinases for differentially regulated phosphosites under acute or chronic stimulation of PDGFRα were performed using Python programming.

Bibliography related to the PDGFR maps

1 Werry, T. D., Wilkinson, G. F. & Willars, G. B. Mechanisms of cross-talk between G-protein-coupled receptors resulting in enhanced release of intracellular Ca2+. *Biochem J* **374**, 281-296, doi:10.1042/BJ20030312 (2003).

2 Wojcikiewicz, R. J. & Luo, S. G. Phosphorylation of inositol 1,4,5-trisphosphate receptors by cAMP-dependent protein kinase. Type I, II, and III receptors are differentially susceptible to phosphorylation and are phosphorylated in intact cells. *J Biol Chem* **273**, 5670-5677 (1998).

3 Bruce, J. I., Shuttleworth, T. J., Giovannucci, D. R. & Yule, D. I. Phosphorylation of inositol 1,4,5-trisphosphate receptors in parotid acinar cells. A mechanism for the synergistic effects of cAMP on Ca2+ signaling. *J Biol Chem* **277**, 1340-1348, doi:10.1074/jbc.M106609200 (2002).

4 Danila, C. I. & Hamilton, S. L. Phosphorylation of ryanodine receptors. *Biol Res* **37**, 521-525 (2004).

5 Okamoto, H. & Takasawa, S. Recent advances in the Okamoto model: the CD38-cyclic ADP-ribose signal system and the regenerating gene protein (Reg)-Reg receptor system in beta-cells. *Diabetes* **51 Suppl 3**, S462-473 (2002).

6 Noguchi, N. *et al.* Cyclic ADP-ribose binds to FK506-binding protein 12.6 to release Ca2+ from islet microsomes. *J Biol Chem* **272**, 3133-3136 (1997).

7 Jang, M. J. *et al.* Phosphorylation of phospholipase D1 and the modulation of its interaction with RhoA by cAMP-dependent protein kinase. *Exp Mol Med* **36**, 172-178, doi:10.1038/emm.2004.24 (2004).

8 Nishizuka, Y. Studies and perspectives of protein kinase C. *Science* **233**, 305-312 (1986).

9 Carpenter, G. & Ji, Q. Phospholipase C-gamma as a signal-transducing element. *Exp Cell Res* **253**, 15-24, doi:10.1006/excr.1999.4671 (1999).

10 Grant, B. D. & Donaldson, J. G. Pathways and mechanisms of endocytic recycling. *Nat Rev Mol Cell Biol* **10**, 597-608, doi:10.1038/nrm2755 (2009).

11 Toschi, A. *et al.* Regulation of mTORC1 and mTORC2 complex assembly by phosphatidic acid: competition with rapamycin. *Mol Cell Biol* **29**, 1411-1420, doi:10.1128/MCB.00782-08 (2009).

12 Thedieck, K. *et al.* PRAS40 and PRR5-like protein are new mTOR interactors that regulate apoptosis. *PLoS One* **2**, e1217, doi:10.1371/journal.pone.0001217 (2007).

13 Peterson, T. R. *et al.* DEPTOR is an mTOR inhibitor frequently overexpressed in multiple myeloma cells and required for their survival. *Cell* **137**, 873-886, doi:10.1016/j.cell.2009.03.046 (2009).

14 Panasyuk, G. *et al.* Nuclear export of S6K1 II is regulated by protein kinase CK2 phosphorylation at Ser-17. *J Biol Chem* **281**, 31188-31201, doi:10.1074/jbc.M602618200 (2006).

15 Hay, N. & Sonenberg, N. Upstream and downstream of mTOR. *Genes Dev* **18**, 1926-1945, doi:10.1101/gad.1212704 (2004).

16 Gingras, A. C., Raught, B. & Sonenberg, N. mTOR signaling to translation. *Curr Top Microbiol Immunol* **279**, 169-197 (2004).

17 Wang, X., Beugnet, A., Murakami, M., Yamanaka, S. & Proud, C. G. Distinct signaling events downstream of mTOR cooperate to mediate the effects of amino acids and

insulin on initiation factor 4E-binding proteins. *Mol Cell Biol* **25**, 2558-2572, doi:10.1128/MCB.25.7.2558-2572.2005 (2005).
18 Wang, X., Li, W., Parra, J. L., Beugnet, A. & Proud, C. G. The C terminus of initiation factor 4E-binding protein 1 contains multiple regulatory features that influence its function and phosphorylation. *Mol Cell Biol* **23**, 1546-1557 (2003).
19 Ferguson, G., Mothe-Satney, I. & Lawrence, J. C., Jr. Ser-64 and Ser-111 in PHAS-I are dispensable for insulin-stimulated dissociation from eIF4E. *J Biol Chem* **278**, 47459-47465, doi:10.1074/jbc.M307949200 (2003).
20 Holz, M. K., Ballif, B. A., Gygi, S. P. & Blenis, J. mTOR and S6K1 mediate assembly of the translation preinitiation complex through dynamic protein interchange and ordered phosphorylation events. *Cell* **123**, 569-580, doi:10.1016/j.cell.2005.10.024 (2005).
21 Ma, X. M. & Blenis, J. Molecular mechanisms of mTOR-mediated translational control. *Nat Rev Mol Cell Biol* **10**, 307-318, doi:10.1038/nrm2672 (2009).
22 Browne, G. J. & Proud, C. G. A novel mTOR-regulated phosphorylation site in elongation factor 2 kinase modulates the activity of the kinase and its binding to calmodulin. *Mol Cell Biol* **24**, 2986-2997 (2004).
23 Redpath, N. T. & Proud, C. G. Cyclic AMP-dependent protein kinase phosphorylates rabbit reticulocyte elongation factor-2 kinase and induces calcium-independent activity. *Biochem J* **293 (Pt 1)**, 31-34 (1993).
24 Diggle, T. A. *et al.* Phosphorylation of elongation factor-2 kinase on serine 499 by cAMP-dependent protein kinase induces Ca2+/calmodulin-independent activity. *Biochem J* **353**, 621-626 (2001).
25 Proud, C. G. Signalling to translation: how signal transduction pathways control the protein synthetic machinery. *Biochem J* **403**, 217-234, doi:10.1042/BJ20070024 (2007).
26 Jimenez, C., Hernandez, C., Pimentel, B. & Carrera, A. C. The p85 regulatory subunit controls sequential activation of phosphoinositide 3-kinase by Tyr kinases and Ras. *J Biol Chem* **277**, 41556-41562, doi:10.1074/jbc.M205893200 (2002).
27 Hooshmand-Rad, R. *et al.* The PI 3-kinase isoforms p110(alpha) and p110(beta) have differential roles in PDGF- and insulin-mediated signaling. *J Cell Sci* **113 Pt 2**, 207-214 (2000).
28 Rinner, O. *et al.* An integrated mass spectrometric and computational framework for the analysis of protein interaction networks. *Nat Biotechnol* **25**, 345-352, doi:10.1038/nbt1289 (2007).
29 Brunet, A. *et al.* Akt promotes cell survival by phosphorylating and inhibiting a Forkhead transcription factor. *Cell* **96**, 857-868 (1999).
30 Li, J., Tewari, M., Vidal, M. & Lee, S. S. The 14-3-3 protein FTT-2 regulates DAF-16 in Caenorhabditis elegans. *Dev Biol* **301**, 82-91, doi:10.1016/j.ydbio.2006.10.013 (2007).
31 Obsilova, V. *et al.* 14-3-3 Protein interacts with nuclear localization sequence of forkhead transcription factor FoxO4. *Biochemistry* **44**, 11608-11617, doi:10.1021/bi050618r (2005).
32 Noel, L. A. *et al.* The tyrosine phosphatase SHP2 is required for cell transformation by the receptor tyrosine kinase mutants FIP1L1-PDGFRalpha and PDGFRalpha D842V. *Mol Oncol* **8**, 728-740, doi:10.1016/j.molonc.2014.02.003 (2014).
33 Feng, H. *et al.* Dynamin 2 mediates PDGFRalpha-SHP-2-promoted glioblastoma growth and invasion. *Oncogene* **31**, 2691-2702, doi:10.1038/onc.2011.436 (2012).
34 Yokote, K., Margolis, B., Heldin, C. H. & Claesson-Welsh, L. Grb7 is a downstream signaling component of platelet-derived growth factor alpha- and beta-receptors. *J Biol Chem* **271**, 30942-30949 (1996).
35 Deakin, N. O. & Turner, C. E. Paxillin comes of age. *J Cell Sci* **121**, 2435-2444, doi:10.1242/jcs.018044 (2008).

36 Brown, M. C. & Turner, C. E. Paxillin: adapting to change. *Physiol Rev* **84**, 1315-1339, doi:10.1152/physrev.00002.2004 (2004).
37 Yokote, K. *et al.* Identification of Tyr-762 in the platelet-derived growth factor alpha-receptor as the binding site for Crk proteins. *Oncogene* **16**, 1229-1239, doi:10.1038/sj.onc.1201641 (1998).
38 Heldin, C. H., Ostman, A. & Ronnstrand, L. Signal transduction via platelet-derived growth factor receptors. *Biochim Biophys Acta* **1378**, F79-113 (1998).
39 Bazenet, C. E., Gelderloos, J. A. & Kazlauskas, A. Phosphorylation of tyrosine 720 in the platelet-derived growth factor alpha receptor is required for binding of Grb2 and SHP-2 but not for activation of Ras or cell proliferation. *Mol Cell Biol* **16**, 6926-6936 (1996).
40 Hooshmand-Rad, R., Yokote, K., Heldin, C. H. & Claesson-Welsh, L. PDGF alpha-receptor mediated cellular responses are not dependent on Src family kinases in endothelial cells. *J Cell Sci* **111 (Pt 5)**, 607-614 (1998).
41 Bai, X. *et al.* Rheb activates mTOR by antagonizing its endogenous inhibitor, FKBP38. *Science* **318**, 977-980, doi:10.1126/science.1147379 (2007).
42 Vander Haar, E., Lee, S. I., Bandhakavi, S., Griffin, T. J. & Kim, D. H. Insulin signalling to mTOR mediated by the Akt/PKB substrate PRAS40. *Nat Cell Biol* **9**, 316-323, doi:10.1038/ncb1547 (2007).
43 Wang, L., Harris, T. E. & Lawrence, J. C., Jr. Regulation of proline-rich Akt substrate of 40 kDa (PRAS40) function by mammalian target of rapamycin complex 1 (mTORC1)-mediated phosphorylation. *J Biol Chem* **283**, 15619-15627, doi:10.1074/jbc.M800723200 (2008).
44 Sancak, Y. *et al.* PRAS40 is an insulin-regulated inhibitor of the mTORC1 protein kinase. *Mol Cell* **25**, 903-915, doi:10.1016/j.molcel.2007.03.003 (2007).
45 Bernardi, R. *et al.* PML inhibits HIF-1alpha translation and neoangiogenesis through repression of mTOR. *Nature* **442**, 779-785, doi:10.1038/nature05029 (2006).
46 Li, Y. *et al.* Bnip3 mediates the hypoxia-induced inhibition on mammalian target of rapamycin by interacting with Rheb. *J Biol Chem* **282**, 35803-35813, doi:10.1074/jbc.M705231200 (2007).
47 Jamieson, J. S. *et al.* Paxillin is essential for PTP-PEST-dependent regulation of cell spreading and motility: a role for paxillin kinase linker. *J Cell Sci* **118**, 5835-5847, doi:10.1242/jcs.02693 (2005).
48 Tsubouchi, A. *et al.* Localized suppression of RhoA activity by Tyr31/118-phosphorylated paxillin in cell adhesion and migration. *J Cell Biol* **159**, 673-683, doi:10.1083/jcb.200202117 (2002).
49 Birge, R. B. *et al.* Identification and characterization of a high-affinity interaction between v-Crk and tyrosine-phosphorylated paxillin in CT10-transformed fibroblasts. *Mol Cell Biol* **13**, 4648-4656 (1993).
50 Petit, V. *et al.* Phosphorylation of tyrosine residues 31 and 118 on paxillin regulates cell migration through an association with CRK in NBT-II cells. *J Cell Biol* **148**, 957-970 (2000).
51 Valles, A. M., Beuvin, M. & Boyer, B. Activation of Rac1 by paxillin-Crk-DOCK180 signaling complex is antagonized by Rap1 in migrating NBT-II cells. *J Biol Chem* **279**, 44490-44496, doi:10.1074/jbc.M405144200 (2004).
52 Moran, M. F., Polakis, P., McCormick, F., Pawson, T. & Ellis, C. Protein-tyrosine kinases regulate the phosphorylation, protein interactions, subcellular distribution, and activity of p21ras GTPase-activating protein. *Mol Cell Biol* **11**, 1804-1812 (1991).
53 Hu, K. Q. & Settleman, J. Tandem SH2 binding sites mediate the RasGAP-RhoGAP interaction: a conformational mechanism for SH3 domain regulation. *EMBO J* **16**, 473-483, doi:10.1093/emboj/16.3.473 (1997).

54 Bryant, S. S. *et al.* Two SH2 domains of p120 Ras GTPase-activating protein bind synergistically to tyrosine phosphorylated p190 Rho GTPase-activating protein. *J Biol Chem* **270**, 17947-17952 (1995).
55 Settleman, J., Albright, C. F., Foster, L. C. & Weinberg, R. A. Association between GTPase activators for Rho and Ras families. *Nature* **359**, 153-154, doi:10.1038/359153a0 (1992).
56 Manes, S. *et al.* Concerted activity of tyrosine phosphatase SHP-2 and focal adhesion kinase in regulation of cell motility. *Mol Cell Biol* **19**, 3125-3135 (1999).
57 Shen, Y., Schneider, G., Cloutier, J. F., Veillette, A. & Schaller, M. D. Direct association of protein-tyrosine phosphatase PTP-PEST with paxillin. *J Biol Chem* **273**, 6474-6481 (1998).
58 Nayal, A. *et al.* Paxillin phosphorylation at Ser273 localizes a GIT1-PIX-PAK complex and regulates adhesion and protrusion dynamics. *J Cell Biol* **173**, 587-589, doi:10.1083/jcb.200509075 (2006).
59 Zhao, Z. S., Manser, E. & Lim, L. Interaction between PAK and nck: a template for Nck targets and role of PAK autophosphorylation. *Mol Cell Biol* **20**, 3906-3917 (2000).
60 Chan, P. M., Lim, L. & Manser, E. PAK is regulated by PI3K, PIX, CDC42, and PP2Calpha and mediates focal adhesion turnover in the hyperosmotic stress-induced p38 pathway. *J Biol Chem* **283**, 24949-24961, doi:10.1074/jbc.M801728200 (2008).
61 Hu, Y., Bock, G., Wick, G. & Xu, Q. Activation of PDGF receptor alpha in vascular smooth muscle cells by mechanical stress. *FASEB J* **12**, 1135-1142 (1998).
62 Vidal, M. *et al.* Molecular and cellular analysis of Grb2 SH3 domain mutants: interaction with Sos and dynamin. *J Mol Biol* **290**, 717-730, doi:10.1006/jmbi.1999.2899 (1999).
63 Morrison, D. K., Kaplan, D. R., Rhee, S. G. & Williams, L. T. Platelet-derived growth factor (PDGF)-dependent association of phospholipase C-gamma with the PDGF receptor signaling complex. *Mol Cell Biol* **10**, 2359-2366 (1990).
64 Ishida, A., Shigeri, Y., Taniguchi, T. & Kameshita, I. Protein phosphatases that regulate multifunctional Ca2+/calmodulin-dependent protein kinases: from biochemistry to pharmacology. *Pharmacol Ther* **100**, 291-305 (2003).
65 Sorimachi, H., Ishiura, S. & Suzuki, K. Structure and physiological function of calpains. *Biochem J* **328 (Pt 3)**, 721-732 (1997).
66 Fayard, E., Tintignac, L. A., Baudry, A. & Hemmings, B. A. Protein kinase B/Akt at a glance. *J Cell Sci* **118**, 5675-5678, doi:10.1242/jcs.02724 (2005).
67 Geltz, N. R. & Augustine, J. A. The p85 and p110 subunits of phosphatidylinositol 3-kinase-alpha are substrates, in vitro, for a constitutively associated protein tyrosine kinase in platelets. *Blood* **91**, 930-939 (1998).
68 Crowley, M. R., Bowtell, D. & Serra, R. TGF-beta, c-Cbl, and PDGFR-alpha the in mammary stroma. *Dev Biol* **279**, 58-72, doi:10.1016/j.ydbio.2004.11.034 (2005).
69 Kikuchi, A. & Monga, S. P. PDGFRalpha in liver pathophysiology: emerging roles in development, regeneration, fibrosis, and cancer. *Gene Expr* **16**, 109-127, doi:10.3727/105221615X14181438356210 (2015).
70 Martin, J., Masri, J., Bernath, A., Nishimura, R. N. & Gera, J. Hsp70 associates with Rictor and is required for mTORC2 formation and activity. *Biochem Biophys Res Commun* **372**, 578-583, doi:10.1016/j.bbrc.2008.05.086 (2008).
71 Frias, M. A. *et al.* mSin1 is necessary for Akt/PKB phosphorylation, and its isoforms define three distinct mTORC2s. *Curr Biol* **16**, 1865-1870, doi:10.1016/j.cub.2006.08.001 (2006).
72 Jacinto, E. What controls TOR? *IUBMB Life* **60**, 483-496, doi:10.1002/iub.56 (2008).
73 Sarbassov, D. D. *et al.* Prolonged rapamycin treatment inhibits mTORC2 assembly and Akt/PKB. *Mol Cell* **22**, 159-168, doi:10.1016/j.molcel.2006.03.029 (2006).

74 Woo, S. Y. *et al.* PRR5, a novel component of mTOR complex 2, regulates platelet-derived growth factor receptor beta expression and signaling. *J Biol Chem* **282**, 25604-25612, doi:10.1074/jbc.M704343200 (2007).
75 Pearce, L. R. *et al.* Identification of Protor as a novel Rictor-binding component of mTOR complex-2. *Biochem J* **405**, 513-522, doi:10.1042/BJ20070540 (2007).
76 Li, Y., Inoki, K., Vacratsis, P. & Guan, K. L. The p38 and MK2 kinase cascade phosphorylates tuberin, the tuberous sclerosis 2 gene product, and enhances its interaction with 14-3-3. *J Biol Chem* **278**, 13663-13671, doi:10.1074/jbc.M300862200 (2003).
77 Sabers, C. J. *et al.* Isolation of a protein target of the FKBP12-rapamycin complex in mammalian cells. *J Biol Chem* **270**, 815-822 (1995).
78 Gwinn, D. M. *et al.* AMPK phosphorylation of raptor mediates a metabolic checkpoint. *Mol Cell* **30**, 214-226, doi:10.1016/j.molcel.2008.03.003 (2008).
79 Currie, R. A. *et al.* Role of phosphatidylinositol 3,4,5-trisphosphate in regulating the activity and localization of 3-phosphoinositide-dependent protein kinase-1. *Biochem J* **337 (Pt 3)**, 575-583 (1999).
80 Galan, J. A. *et al.* Phosphoproteomic analysis identifies the tumor suppressor PDCD4 as a RSK substrate negatively regulated by 14-3-3. *Proc Natl Acad Sci U S A* **111**, E2918-2927, doi:10.1073/pnas.1405601111 (2014).
81 Michlewski, G., Sanford, J. R. & Caceres, J. F. The splicing factor SF2/ASF regulates translation initiation by enhancing phosphorylation of 4E-BP1. *Mol Cell* **30**, 179-189, doi:10.1016/j.molcel.2008.03.013 (2008).
82 Nojima, T., Hirose, T., Kimura, H. & Hagiwara, M. The interaction between cap-binding complex and RNA export factor is required for intronless mRNA export. *J Biol Chem* **282**, 15645-15651, doi:10.1074/jbc.M700629200 (2007).
83 Ma, X. M., Yoon, S. O., Richardson, C. J., Julich, K. & Blenis, J. SKAR links pre-mRNA splicing to mTOR/S6K1-mediated enhanced translation efficiency of spliced mRNAs. *Cell* **133**, 303-313, doi:10.1016/j.cell.2008.02.031 (2008).
84 Le Hir, H. & Seraphin, B. EJCs at the heart of translational control. *Cell* **133**, 213-216, doi:10.1016/j.cell.2008.04.002 (2008).
85 Richardson, C. J. *et al.* SKAR is a specific target of S6 kinase 1 in cell growth control. *Curr Biol* **14**, 1540-1549, doi:10.1016/j.cub.2004.08.061 (2004).
86 Ling, J., Morley, S. J. & Traugh, J. A. Inhibition of cap-dependent translation via phosphorylation of eIF4G by protein kinase Pak2. *EMBO J* **24**, 4094-4105, doi:10.1038/sj.emboj.7600868 (2005).
87 Yang, H. S. *et al.* The transformation suppressor Pdcd4 is a novel eukaryotic translation initiation factor 4A binding protein that inhibits translation. *Mol Cell Biol* **23**, 26-37 (2003).
88 Yang, H. S. *et al.* A novel function of the MA-3 domains in transformation and translation suppressor Pdcd4 is essential for its binding to eukaryotic translation initiation factor 4A. *Mol Cell Biol* **24**, 3894-3906 (2004).
89 Wilker, E. W. *et al.* 14-3-3sigma controls mitotic translation to facilitate cytokinesis. *Nature* **446**, 329-332, doi:10.1038/nature05584 (2007).
90 Mitsui, K., Brady, M., Palfrey, H. C. & Nairn, A. C. Purification and characterization of calmodulin-dependent protein kinase III from rabbit reticulocytes and rat pancreas. *J Biol Chem* **268**, 13422-13433 (1993).
91 Redpath, N. T. & Proud, C. G. Purification and phosphorylation of elongation factor-2 kinase from rabbit reticulocytes. *Eur J Biochem* **212**, 511-520 (1993).
92 Gulati, P. *et al.* Amino acids activate mTOR complex 1 via Ca2+/CaM signaling to hVps34. *Cell Metab* **7**, 456-465, doi:10.1016/j.cmet.2008.03.002 (2008).
93 Carlberg, U., Nilsson, A. & Nygard, O. Functional properties of phosphorylated elongation factor 2. *Eur J Biochem* **191**, 639-645 (1990).

94 Mader, S., Lee, H., Pause, A. & Sonenberg, N. The translation initiation factor eIF-4E binds to a common motif shared by the translation factor eIF-4 gamma and the translational repressors 4E-binding proteins. *Mol Cell Biol* **15**, 4990-4997 (1995).
95 Gingras, A. C., Raught, B. & Sonenberg, N. eIF4 initiation factors: effectors of mRNA recruitment to ribosomes and regulators of translation. *Annu Rev Biochem* **68**, 913-963, doi:10.1146/annurev.biochem.68.1.913 (1999).
96 Gan, B., Melkoumian, Z. K., Wu, X., Guan, K. L. & Guan, J. L. Identification of FIP200 interaction with the TSC1-TSC2 complex and its role in regulation of cell size control. *J Cell Biol* **170**, 379-389, doi:10.1083/jcb.200411106 (2005).
97 Wei, Q., Miskimins, W. K. & Miskimins, R. Sox10 acts as a tissue-specific transcription factor enhancing activation of the myelin basic protein gene promoter by p27Kip1 and Sp1. *J Neurosci Res* **78**, 796-802, doi:10.1002/jnr.20342 (2004).
98 Chew, L. J., Coley, W., Cheng, Y. & Gallo, V. Mechanisms of regulation of oligodendrocyte development by p38 mitogen-activated protein kinase. *J Neurosci* **30**, 11011-11027, doi:10.1523/JNEUROSCI.2546-10.2010 (2010).
99 Azahri, N. S., Di Bartolo, B. A., Khachigian, L. M. & Kavurma, M. M. Sp1, acetylated histone-3 and p300 regulate TRAIL transcription: mechanisms of PDGF-BB-mediated VSMC proliferation and migration. *J Cell Biochem* **113**, 2597-2606, doi:10.1002/jcb.24135 (2012).
100 Minato, Y. *et al.* Transcriptional regulation of a new variant of human platelet-derived growth factor receptor alpha transcript by E2F-1. *Gene* **403**, 89-97, doi:10.1016/j.gene.2007.08.011 (2007).
101 Liu, M. Y., Eyries, M., Zhang, C., Santiago, F. S. & Khachigian, L. M. Inducible platelet-derived growth factor D-chain expression by angiotensin II and hydrogen peroxide involves transcriptional regulation by Ets-1 and Sp1. *Blood* **107**, 2322-2329, doi:10.1182/blood-2005-06-2377 (2006).
102 Sanchez-Guerrero, E., Midgley, V. C. & Khachigian, L. M. Angiotensin II induction of PDGF-C expression is mediated by AT1 receptor-dependent Egr-1 transactivation. *Nucleic Acids Res* **36**, 1941-1951, doi:10.1093/nar/gkm923 (2008).
103 Rafty, L. A. & Khachigian, L. M. Sp1 phosphorylation regulates inducible expression of platelet-derived growth factor B-chain gene via atypical protein kinase C-zeta. *Nucleic Acids Res* **29**, 1027-1033 (2001).
104 Rafty, L. A., Santiago, F. S. & Khachigian, L. M. NF1/X represses PDGF A-chain transcription by interacting with Sp1 and antagonizing Sp1 occupancy of the promoter. *EMBO J* **21**, 334-343, doi:10.1093/emboj/21.3.334 (2002).
105 Xie, J. *et al.* A role of PDGFRalpha in basal cell carcinoma proliferation. *Proc Natl Acad Sci U S A* **98**, 9255-9259, doi:10.1073/pnas.151173398 (2001).
106 Palomero, J. *et al.* SOX11 promotes tumor angiogenesis through transcriptional regulation of PDGFA in mantle cell lymphoma. *Blood* **124**, 2235-2247, doi:10.1182/blood-2014-04-569566 (2014).
107 Joosten, P. H. *et al.* Altered regulation of platelet-derived growth factor receptor-alpha gene-transcription in vitro by spina bifida-associated mutant Pax1 proteins. *Proc Natl Acad Sci U S A* **95**, 14459-14463 (1998).
108 Zhang, N., Chan, C. W., Sanchez-Guerrero, E. & Khachigian, L. M. Repression of PDGF-R-alpha after cellular injury involves TNF-alpha, formation of a c-Fos-YY1 complex, and negative regulation by HDAC. *Am J Physiol Cell Physiol* **302**, C1590-1598, doi:10.1152/ajpcell.00429.2011 (2012).
109 Meng, F. *et al.* PDGFRalpha and beta play critical roles in mediating Foxq1-driven breast cancer stemness and chemoresistance. *Cancer Res* **75**, 584-593, doi:10.1158/0008-5472.CAN-13-3029 (2015).

110 Pereira, L. A. *et al.* Pdgfralpha and Flk1 are direct target genes of Mixl1 in differentiating embryonic stem cells. *Stem Cell Res* **8**, 165-179, doi:10.1016/j.scr.2011.09.007 (2012).
111 Wang, C. & Song, B. Cell-type-specific expression of the platelet-derived growth factor alpha receptor: a role for GATA-binding protein. *Mol Cell Biol* **16**, 712-723 (1996).
112 Bonello, M. R. & Khachigian, L. M. Fibroblast growth factor-2 represses platelet-derived growth factor receptor-alpha (PDGFR-alpha) transcription via ERK1/2-dependent Sp1 phosphorylation and an atypical cis-acting element in the proximal PDGFR-alpha promoter. *J Biol Chem* **279**, 2377-2382, doi:10.1074/jbc.M308254200 (2004).
113 Schreiber, M. *et al.* Control of cell cycle progression by c-Jun is p53 dependent. *Genes Dev* **13**, 607-619 (1999).
114 Ueno, Y. *et al.* Lysophosphatidylcholine phosphorylates CREB and activates the jun2TRE site of c-jun promoter in vascular endothelial cells. *FEBS Lett* **457**, 241-245 (1999).
115 Warner, B. J., Blain, S. W., Seoane, J. & Massague, J. Myc downregulation by transforming growth factor beta required for activation of the p15(Ink4b) G(1) arrest pathway. *Mol Cell Biol* **19**, 5913-5922 (1999).
116 Gartel, A. L. & Shchors, K. Mechanisms of c-myc-mediated transcriptional repression of growth arrest genes. *Exp Cell Res* **283**, 17-21 (2003).
117 Claassen, G. F. & Hann, S. R. A role for transcriptional repression of p21CIP1 by c-Myc in overcoming transforming growth factor beta -induced cell-cycle arrest. *Proc Natl Acad Sci U S A* **97**, 9498-9503, doi:10.1073/pnas.150006697 (2000).
118 Kishi, H. *et al.* Osmotic shock induces G1 arrest through p53 phosphorylation at Ser33 by activated p38MAPK without phosphorylation at Ser15 and Ser20. *J Biol Chem* **276**, 39115-39122, doi:10.1074/jbc.M105134200 (2001).
119 Sato, K., Nagao, T., Iwasaki, T., Nishihira, Y. & Fukami, Y. Src-dependent phosphorylation of the EGF receptor Tyr-845 mediates Stat-p21waf1 pathway in A431 cells. *Genes Cells* **8**, 995-1003 (2003).
120 Weisz, A. & Rosales, R. Identification of an estrogen response element upstream of the human c-fos gene that binds the estrogen receptor and the AP-1 transcription factor. *Nucleic Acids Res* **18**, 5097-5106 (1990).
121 Calnan, D. R. & Brunet, A. The FoxO code. *Oncogene* **27**, 2276-2288, doi:10.1038/onc.2008.21 (2008).
122 Shtutman, M. *et al.* The cyclin D1 gene is a target of the beta-catenin/LEF-1 pathway. *Proc Natl Acad Sci U S A* **96**, 5522-5527 (1999).
123 Tokino, T. & Nakamura, Y. The role of p53-target genes in human cancer. *Crit Rev Oncol Hematol* **33**, 1-6 (2000).
124 Levine, A. J., Feng, Z., Mak, T. W., You, H. & Jin, S. Coordination and communication between the p53 and IGF-1-AKT-TOR signal transduction pathways. *Genes Dev* **20**, 267-275, doi:10.1101/gad.1363206 (2006).
125 Greer, E. L. *et al.* The energy sensor AMP-activated protein kinase directly regulates the mammalian FOXO3 transcription factor. *J Biol Chem* **282**, 30107-30119, doi:10.1074/jbc.M705325200 (2007).
126 Smith, E. M. & Proud, C. G. cdc2-cyclin B regulates eEF2 kinase activity in a cell cycle- and amino acid-dependent manner. *EMBO J* **27**, 1005-1016, doi:10.1038/emboj.2008.39 (2008).
127 Roig, J. & Traugh, J. A. Cytostatic p21 G protein-activated protein kinase gamma-PAK. *Vitam Horm* **62**, 167-198 (2001).
128 Downward, J. PI 3-kinase, Akt and cell survival. *Semin Cell Dev Biol* **15**, 177-182 (2004).
129 Darnell, J. E., Jr. STATs and gene regulation. *Science* **277**, 1630-1635 (1997).

130 Vignais, M. L. & Gilman, M. Distinct mechanisms of activation of Stat1 and Stat3 by platelet-derived growth factor receptor in a cell-free system. *Mol Cell Biol* **19**, 3727-3735 (1999).
131 Turkson, J. *et al.* Requirement for Ras/Rac1-mediated p38 and c-Jun N-terminal kinase signaling in Stat3 transcriptional activity induced by the Src oncoprotein. *Mol Cell Biol* **19**, 7519-7528 (1999).
132 Valgeirsdottir, S., Paukku, K., Silvennoinen, O., Heldin, C. H. & Claesson-Welsh, L. Activation of Stat5 by platelet-derived growth factor (PDGF) is dependent on phosphorylation sites in PDGF beta-receptor juxtamembrane and kinase insert domains. *Oncogene* **16**, 505-515, doi:10.1038/sj.onc.1201555 (1998).
133 Goh, K. C., Haque, S. J. & Williams, B. R. p38 MAP kinase is required for STAT1 serine phosphorylation and transcriptional activation induced by interferons. *EMBO J* **18**, 5601-5608, doi:10.1093/emboj/18.20.5601 (1999).
134 Vermeulen, L., De Wilde, G., Van Damme, P., Vanden Berghe, W. & Haegeman, G. Transcriptional activation of the NF-kappaB p65 subunit by mitogen- and stress-activated protein kinase-1 (MSK1). *EMBO J* **22**, 1313-1324, doi:10.1093/emboj/cdg139 (2003).
135 Jacks, K. A. & Koch, C. A. Differential regulation of mitogen- and stress-activated protein kinase-1 and -2 (MSK1 and MSK2) by CK2 following UV radiation. *J Biol Chem* **285**, 1661-1670, doi:10.1074/jbc.M109.083808 (2010).
136 Raingeaud, J. *et al.* Pro-inflammatory cytokines and environmental stress cause p38 mitogen-activated protein kinase activation by dual phosphorylation on tyrosine and threonine. *J Biol Chem* **270**, 7420-7426 (1995).
137 Roux, P. P. & Blenis, J. ERK and p38 MAPK-activated protein kinases: a family of protein kinases with diverse biological functions. *Microbiol Mol Biol Rev* **68**, 320-344, doi:10.1128/MMBR.68.2.320-344.2004 (2004).
138 Wiggin, G. R. *et al.* MSK1 and MSK2 are required for the mitogen- and stress-induced phosphorylation of CREB and ATF1 in fibroblasts. *Mol Cell Biol* **22**, 2871-2881 (2002).
139 Heidenreich, O. *et al.* MAPKAP kinase 2 phosphorylates serum response factor in vitro and in vivo. *J Biol Chem* **274**, 14434-14443 (1999).
140 Clifton, A. D., Young, P. R. & Cohen, P. A comparison of the substrate specificity of MAPKAP kinase-2 and MAPKAP kinase-3 and their activation by cytokines and cellular stress. *FEBS Lett* **392**, 209-214 (1996).
141 McCoy, C. E., Campbell, D. G., Deak, M., Bloomberg, G. B. & Arthur, J. S. MSK1 activity is controlled by multiple phosphorylation sites. *Biochem J* **387**, 507-517, doi:10.1042/BJ20041501 (2005).
142 Wang, X. Z. & Ron, D. Stress-induced phosphorylation and activation of the transcription factor CHOP (GADD153) by p38 MAP Kinase. *Science* **272**, 1347-1349 (1996).
143 Foulds, C. E., Nelson, M. L., Blaszczak, A. G. & Graves, B. J. Ras/mitogen-activated protein kinase signaling activates Ets-1 and Ets-2 by CBP/p300 recruitment. *Mol Cell Biol* **24**, 10954-10964, doi:10.1128/MCB.24.24.10954-10964.2004 (2004).
144 Seth, A., Alvarez, E., Gupta, S. & Davis, R. J. A phosphorylation site located in the NH2-terminal domain of c-Myc increases transactivation of gene expression. *J Biol Chem* **266**, 23521-23524 (1991).
145 Alvarez, E. *et al.* Pro-Leu-Ser/Thr-Pro is a consensus primary sequence for substrate protein phosphorylation. Characterization of the phosphorylation of c-myc and c-jun proteins by an epidermal growth factor receptor threonine 669 protein kinase. *J Biol Chem* **266**, 15277-15285 (1991).
146 Gupta, S., Seth, A. & Davis, R. J. Transactivation of gene expression by Myc is inhibited by mutation at the phosphorylation sites Thr-58 and Ser-62. *Proc Natl Acad Sci U S A* **90**, 3216-3220 (1993).

147 Lord, J. D., McIntosh, B. C., Greenberg, P. D. & Nelson, B. H. The IL-2 receptor promotes lymphocyte proliferation and induction of the c-myc, bcl-2, and bcl-x genes through the trans-activation domain of Stat5. *J Immunol* **164**, 2533-2541 (2000).
148 Sun, H., Charles, C. H., Lau, L. F. & Tonks, N. K. MKP-1 (3CH134), an immediate early gene product, is a dual specificity phosphatase that dephosphorylates MAP kinase in vivo. *Cell* **75**, 487-493 (1993).
149 Franklin, C. C. & Kraft, A. S. Constitutively active MAP kinase kinase (MEK1) stimulates SAP kinase and c-Jun transcriptional activity in U937 human leukemic cells. *Oncogene* **11**, 2365-2374 (1995).
150 Tian, J. & Karin, M. Stimulation of Elk1 transcriptional activity by mitogen-activated protein kinases is negatively regulated by protein phosphatase 2B (calcineurin). *J Biol Chem* **274**, 15173-15180 (1999).
151 Cavigelli, M., Dolfi, F., Claret, F. X. & Karin, M. Induction of c-fos expression through JNK-mediated TCF/Elk-1 phosphorylation. *EMBO J* **14**, 5957-5964 (1995).
152 Horgan, A. M. & Stork, P. J. Examining the mechanism of Erk nuclear translocation using green fluorescent protein. *Exp Cell Res* **285**, 208-220 (2003).
153 Marais, R., Wynne, J. & Treisman, R. The SRF accessory protein Elk-1 contains a growth factor-regulated transcriptional activation domain. *Cell* **73**, 381-393 (1993).
154 Aplin, A. E., Stewart, S. A., Assoian, R. K. & Juliano, R. L. Integrin-mediated adhesion regulates ERK nuclear translocation and phosphorylation of Elk-1. *J Cell Biol* **153**, 273-282 (2001).
155 Ginty, D. D., Bonni, A. & Greenberg, M. E. Nerve growth factor activates a Ras-dependent protein kinase that stimulates c-fos transcription via phosphorylation of CREB. *Cell* **77**, 713-725 (1994).
156 Xing, J., Kornhauser, J. M., Xia, Z., Thiele, E. A. & Greenberg, M. E. Nerve growth factor activates extracellular signal-regulated kinase and p38 mitogen-activated protein kinase pathways to stimulate CREB serine 133 phosphorylation. *Mol Cell Biol* **18**, 1946-1955 (1998).
157 De Cesare, D., Jacquot, S., Hanauer, A. & Sassone-Corsi, P. Rsk-2 activity is necessary for epidermal growth factor-induced phosphorylation of CREB protein and transcription of c-fos gene. *Proc Natl Acad Sci U S A* **95**, 12202-12207 (1998).
158 Brunet, A. *et al.* Protein kinase SGK mediates survival signals by phosphorylating the forkhead transcription factor FKHRL1 (FOXO3a). *Mol Cell Biol* **21**, 952-965, doi:10.1128/MCB.21.3.952-965.2001 (2001).
159 Jacinto, E. *et al.* SIN1/MIP1 maintains rictor-mTOR complex integrity and regulates Akt phosphorylation and substrate specificity. *Cell* **127**, 125-137, doi:10.1016/j.cell.2006.08.033 (2006).
160 Jones, R. G. *et al.* AMP-activated protein kinase induces a p53-dependent metabolic checkpoint. *Mol Cell* **18**, 283-293, doi:10.1016/j.molcel.2005.03.027 (2005).
161 Feng, Z., Zhang, H., Levine, A. J. & Jin, S. The coordinate regulation of the p53 and mTOR pathways in cells. *Proc Natl Acad Sci U S A* **102**, 8204-8209, doi:10.1073/pnas.0502857102 (2005).
162 Karuman, P. *et al.* The Peutz-Jegher gene product LKB1 is a mediator of p53-dependent cell death. *Mol Cell* **7**, 1307-1319 (2001).
163 Ahn, S., Maudsley, S., Luttrell, L. M., Lefkowitz, R. J. & Daaka, Y. Src-mediated tyrosine phosphorylation of dynamin is required for beta2-adrenergic receptor internalization and mitogen-activated protein kinase signaling. *J Biol Chem* **274**, 1185-1188 (1999).
164 Piccaluga, P. P. *et al.* Platelet-derived growth factor alpha mediates the proliferation of peripheral T-cell lymphoma cells via an autocrine regulatory pathway. *Leukemia* **28**, 1687-1697, doi:10.1038/leu.2014.50 (2014).

165 Buitenhuis, M., Verhagen, L. P., Cools, J. & Coffer, P. J. Molecular mechanisms underlying FIP1L1-PDGFRA-mediated myeloproliferation. *Cancer Res* **67**, 3759-3766, doi:10.1158/0008-5472.CAN-06-4183 (2007).
166 Karin, M., Liu, Z. & Zandi, E. AP-1 function and regulation. *Curr Opin Cell Biol* **9**, 240-246 (1997).
167 Manning, B. D. & Cantley, L. C. Rheb fills a GAP between TSC and TOR. *Trends Biochem Sci* **28**, 573-576, doi:10.1016/j.tibs.2003.09.003 (2003).
168 Kwiatkowski, D. J. & Manning, B. D. Tuberous sclerosis: a GAP at the crossroads of multiple signaling pathways. *Hum Mol Genet* **14 Spec No. 2**, R251-258, doi:10.1093/hmg/ddi260 (2005).
169 Inoki, K., Li, Y., Xu, T. & Guan, K. L. Rheb GTPase is a direct target of TSC2 GAP activity and regulates mTOR signaling. *Genes Dev* **17**, 1829-1834, doi:10.1101/gad.1110003 (2003).
170 Ma, L., Chen, Z., Erdjument-Bromage, H., Tempst, P. & Pandolfi, P. P. Phosphorylation and functional inactivation of TSC2 by Erk implications for tuberous sclerosis and cancer pathogenesis. *Cell* **121**, 179-193, doi:10.1016/j.cell.2005.02.031 (2005).
171 Ma, L. *et al.* Identification of S664 TSC2 phosphorylation as a marker for extracellular signal-regulated kinase mediated mTOR activation in tuberous sclerosis and human cancer. *Cancer Res* **67**, 7106-7112, doi:10.1158/0008-5472.CAN-06-4798 (2007).
172 Rolfe, M., McLeod, L. E., Pratt, P. F. & Proud, C. G. Activation of protein synthesis in cardiomyocytes by the hypertrophic agent phenylephrine requires the activation of ERK and involves phosphorylation of tuberous sclerosis complex 2 (TSC2). *Biochem J* **388**, 973-984, doi:10.1042/BJ20041888 (2005).
173 Cai, S. L. *et al.* Activity of TSC2 is inhibited by AKT-mediated phosphorylation and membrane partitioning. *J Cell Biol* **173**, 279-289, doi:10.1083/jcb.200507119 (2006).
174 Inoki, K., Li, Y., Zhu, T., Wu, J. & Guan, K. L. TSC2 is phosphorylated and inhibited by Akt and suppresses mTOR signalling. *Nat Cell Biol* **4**, 648-657, doi:10.1038/ncb839 (2002).
175 Potter, C. J., Pedraza, L. G. & Xu, T. Akt regulates growth by directly phosphorylating Tsc2. *Nat Cell Biol* **4**, 658-665, doi:10.1038/ncb840 (2002).
176 Manning, B. D., Tee, A. R., Logsdon, M. N., Blenis, J. & Cantley, L. C. Identification of the tuberous sclerosis complex-2 tumor suppressor gene product tuberin as a target of the phosphoinositide 3-kinase/akt pathway. *Mol Cell* **10**, 151-162 (2002).
177 Dan, H. C. *et al.* Phosphatidylinositol 3-kinase/Akt pathway regulates tuberous sclerosis tumor suppressor complex by phosphorylation of tuberin. *J Biol Chem* **277**, 35364-35370, doi:10.1074/jbc.M205838200 (2002).
178 Theodosiou, A., Smith, A., Gillieron, C., Arkinstall, S. & Ashworth, A. MKP5, a new member of the MAP kinase phosphatase family, which selectively dephosphorylates stress-activated kinases. *Oncogene* **18**, 6981-6988, doi:10.1038/sj.onc.1203185 (1999).
179 Raingeaud, J., Whitmarsh, A. J., Barrett, T., Derijard, B. & Davis, R. J. MKK3- and MKK6-regulated gene expression is mediated by the p38 mitogen-activated protein kinase signal transduction pathway. *Mol Cell Biol* **16**, 1247-1255 (1996).
180 Waskiewicz, A. J., Flynn, A., Proud, C. G. & Cooper, J. A. Mitogen-activated protein kinases activate the serine/threonine kinases Mnk1 and Mnk2. *EMBO J* **16**, 1909-1920, doi:10.1093/emboj/16.8.1909 (1997).
181 Nick, J. A. *et al.* Selective activation and functional significance of p38alpha mitogen-activated protein kinase in lipopolysaccharide-stimulated neutrophils. *J Clin Invest* **103**, 851-858, doi:10.1172/JCI5257 (1999).
182 Tall, G. G., Barbieri, M. A., Stahl, P. D. & Horazdovsky, B. F. Ras-activated endocytosis is mediated by the Rab5 guanine nucleotide exchange activity of RIN1. *Dev Cell* **1**, 73-82 (2001).

183 Schaller, M. D. & Parsons, J. T. pp125FAK-dependent tyrosine phosphorylation of paxillin creates a high-affinity binding site for Crk. *Mol Cell Biol* **15**, 2635-2645 (1995).
184 Jacinto, E. *et al.* Mammalian TOR complex 2 controls the actin cytoskeleton and is rapamycin insensitive. *Nat Cell Biol* **6**, 1122-1128, doi:10.1038/ncb1183 (2004).
185 Jackson, J. L. & Young, M. R. Protein phosphatase-2A regulates protein tyrosine phosphatase activity in Lewis lung carcinoma tumor variants. *Clin Exp Metastasis* **20**, 357-364 (2003).
186 Ito, A. *et al.* A truncated isoform of the PP2A B56 subunit promotes cell motility through paxillin phosphorylation. *EMBO J* **19**, 562-571, doi:10.1093/emboj/19.4.562 (2000).
187 Young, M. R., Liu, S. W. & Meisinger, J. Protein phosphatase-2A restricts migration of Lewis lung carcinoma cells by modulating the phosphorylation of focal adhesion proteins. *Int J Cancer* **103**, 38-44, doi:10.1002/ijc.10772 (2003).
188 Brown, M. C., Cary, L. A., Jamieson, J. S., Cooper, J. A. & Turner, C. E. Src and FAK kinases cooperate to phosphorylate paxillin kinase linker, stimulate its focal adhesion localization, and regulate cell spreading and protrusiveness. *Mol Biol Cell* **16**, 4316-4328, doi:10.1091/mbc.E05-02-0131 (2005).
189 Loo, T. H., Ng, Y. W., Lim, L. & Manser, E. GIT1 activates p21-activated kinase through a mechanism independent of p21 binding. *Mol Cell Biol* **24**, 3849-3859 (2004).
190 Lei, M. *et al.* Structure of PAK1 in an autoinhibited conformation reveals a multistage activation switch. *Cell* **102**, 387-397 (2000).
191 Huang, C., Rajfur, Z., Borchers, C., Schaller, M. D. & Jacobson, K. JNK phosphorylates paxillin and regulates cell migration. *Nature* **424**, 219-223, doi:10.1038/nature01745 (2003).
192 Kishimoto, H. *et al.* Different properties of SEK1 and MKK7 in dual phosphorylation of stress-induced activated protein kinase SAPK/JNK in embryonic stem cells. *J Biol Chem* **278**, 16595-16601, doi:10.1074/jbc.M213182200 (2003).
193 Yujiri, T., Sather, S., Fanger, G. R. & Johnson, G. L. Role of MEKK1 in cell survival and activation of JNK and ERK pathways defined by targeted gene disruption. *Science* **282**, 1911-1914 (1998).
194 Moriguchi, T. *et al.* A novel SAPK/JNK kinase, MKK7, stimulated by TNFalpha and cellular stresses. *EMBO J* **16**, 7045-7053, doi:10.1093/emboj/16.23.7045 (1997).
195 Yao, Z. *et al.* Activation of stress-activated protein kinases/c-Jun N-terminal protein kinases (SAPKs/JNKs) by a novel mitogen-activated protein kinase kinase. *J Biol Chem* **272**, 32378-32383 (1997).
196 Lu, X., Nemoto, S. & Lin, A. Identification of c-Jun NH2-terminal protein kinase (JNK)-activating kinase 2 as an activator of JNK but not p38. *J Biol Chem* **272**, 24751-24754 (1997).
197 Takahashi, H. *et al.* Expression of human cystatin A by keratinocytes is positively regulated via the Ras/MEKK1/MKK7/JNK signal transduction pathway but negatively regulated via the Ras/Raf-1/MEK1/ERK pathway. *J Biol Chem* **276**, 36632-36638, doi:10.1074/jbc.M102021200 (2001).
198 Tibbles, L. A. *et al.* MLK-3 activates the SAPK/JNK and p38/RK pathways via SEK1 and MKK3/6. *EMBO J* **15**, 7026-7035 (1996).
199 Ichijo, H. *et al.* Induction of apoptosis by ASK1, a mammalian MAPKKK that activates SAPK/JNK and p38 signaling pathways. *Science* **275**, 90-94 (1997).
200 Moriguchi, T. *et al.* A novel kinase cascade mediated by mitogen-activated protein kinase kinase 6 and MKK3. *J Biol Chem* **271**, 13675-13679 (1996).
201 Burbelo, P. D., Drechsel, D. & Hall, A. A conserved binding motif defines numerous candidate target proteins for both Cdc42 and Rac GTPases. *J Biol Chem* **270**, 29071-29074 (1995).

202 Fanger, G. R., Johnson, N. L. & Johnson, G. L. MEK kinases are regulated by EGF and selectively interact with Rac/Cdc42. *EMBO J* **16**, 4961-4972, doi:10.1093/emboj/16.16.4961 (1997).
203 Chi, H., Sarkisian, M. R., Rakic, P. & Flavell, R. A. Loss of mitogen-activated protein kinase kinase kinase 4 (MEKK4) results in enhanced apoptosis and defective neural tube development. *Proc Natl Acad Sci U S A* **102**, 3846-3851, doi:10.1073/pnas.0500026102 (2005).
204 Witowsky, J. A. & Johnson, G. L. Ubiquitylation of MEKK1 inhibits its phosphorylation of MKK1 and MKK4 and activation of the ERK1/2 and JNK pathways. *J Biol Chem* **278**, 1403-1406, doi:10.1074/jbc.C200616200 (2003).
205 Guan, Z., Buckman, S. Y., Pentland, A. P., Templeton, D. J. & Morrison, A. R. Induction of cyclooxygenase-2 by the activated MEKK1 --> SEK1/MKK4 --> p38 mitogen-activated protein kinase pathway. *J Biol Chem* **273**, 12901-12908 (1998).
206 Kolch, W. Meaningful relationships: the regulation of the Ras/Raf/MEK/ERK pathway by protein interactions. *Biochem J* **351 Pt 2**, 289-305 (2000).
207 Kyriakis, J. M. *et al.* Raf-1 activates MAP kinase-kinase. *Nature* **358**, 417-421, doi:10.1038/358417a0 (1992).
208 Dent, P. *et al.* Activation of mitogen-activated protein kinase kinase by v-Raf in NIH 3T3 cells and in vitro. *Science* **257**, 1404-1407 (1992).
209 Wu, J. *et al.* Identification and characterization of a new mammalian mitogen-activated protein kinase kinase, MKK2. *Mol Cell Biol* **13**, 4539-4548 (1993).
210 Lange-Carter, C. A., Pleiman, C. M., Gardner, A. M., Blumer, K. J. & Johnson, G. L. A divergence in the MAP kinase regulatory network defined by MEK kinase and Raf. *Science* **260**, 315-319 (1993).
211 Xu, S. *et al.* MEKK1 phosphorylates MEK1 and MEK2 but does not cause activation of mitogen-activated protein kinase. *Proc Natl Acad Sci U S A* **92**, 6808-6812 (1995).
212 Lawler, S., Cuenda, A., Goedert, M. & Cohen, P. SKK4, a novel activator of stress-activated protein kinase-1 (SAPK1/JNK). *FEBS Lett* **414**, 153-158 (1997).
213 Lu, Z., Xu, S., Joazeiro, C., Cobb, M. H. & Hunter, T. The PHD domain of MEKK1 acts as an E3 ubiquitin ligase and mediates ubiquitination and degradation of ERK1/2. *Mol Cell* **9**, 945-956 (2002).
214 Cox, A. D. & Der, C. J. The dark side of Ras: regulation of apoptosis. *Oncogene* **22**, 8999-9006, doi:10.1038/sj.onc.1207111 (2003).
215 Diekmann, D. *et al.* Bcr encodes a GTPase-activating protein for p21rac. *Nature* **351**, 400-402, doi:10.1038/351400a0 (1991).
216 Ahmed, S. *et al.* A novel functional target for tumor-promoting phorbol esters and lysophosphatidic acid. The p21rac-GTPase activating protein n-chimaerin. *J Biol Chem* **268**, 10709-10712 (1993).
217 Caloca, M. J., Wang, H., Delemos, A., Wang, S. & Kazanietz, M. G. Phorbol esters and related analogs regulate the subcellular localization of beta 2-chimaerin, a non-protein kinase C phorbol ester receptor. *J Biol Chem* **276**, 18303-18312, doi:10.1074/jbc.M011368200 (2001).
218 Sastry, S. K., Lyons, P. D., Schaller, M. D. & Burridge, K. PTP-PEST controls motility through regulation of Rac1. *J Cell Sci* **115**, 4305-4316 (2002).
219 Brugnera, E. *et al.* Unconventional Rac-GEF activity is mediated through the Dock180-ELMO complex. *Nat Cell Biol* **4**, 574-582, doi:10.1038/ncb824 (2002).
220 Grimsley, C. M. *et al.* Dock180 and ELMO1 proteins cooperate to promote evolutionarily conserved Rac-dependent cell migration. *J Biol Chem* **279**, 6087-6097, doi:10.1074/jbc.M307087200 (2004).
221 Cleghon, V. & Morrison, D. K. Raf-1 interacts with Fyn and Src in a non-phosphotyrosine-dependent manner. *J Biol Chem* **269**, 17749-17755 (1994).

222 King, A. J., Wireman, R. S., Hamilton, M. & Marshall, M. S. Phosphorylation site specificity of the Pak-mediated regulation of Raf-1 and cooperativity with Src. *FEBS Lett* **497**, 6-14 (2001).
223 Tran, N. H. & Frost, J. A. Phosphorylation of Raf-1 by p21-activated kinase 1 and Src regulates Raf-1 autoinhibition. *J Biol Chem* **278**, 11221-11226, doi:10.1074/jbc.M210318200 (2003).
224 Mason, C. S. *et al.* Serine and tyrosine phosphorylations cooperate in Raf-1, but not B-Raf activation. *EMBO J* **18**, 2137-2148, doi:10.1093/emboj/18.8.2137 (1999).
225 Zimmermann, S. & Moelling, K. Phosphorylation and regulation of Raf by Akt (protein kinase B). *Science* **286**, 1741-1744 (1999).
226 Reusch, H. P., Zimmermann, S., Schaefer, M., Paul, M. & Moelling, K. Regulation of Raf by Akt controls growth and differentiation in vascular smooth muscle cells. *J Biol Chem* **276**, 33630-33637, doi:10.1074/jbc.M105322200 (2001).
227 Han, L. *et al.* Protein binding and signaling properties of RIN1 suggest a unique effector function. *Proc Natl Acad Sci U S A* **94**, 4954-4959 (1997).
228 Ponting, C. P. & Benjamin, D. R. A novel family of Ras-binding domains. *Trends Biochem Sci* **21**, 422-425 (1996).
229 Li, W. *et al.* A new function for a phosphotyrosine phosphatase: linking GRB2-Sos to a receptor tyrosine kinase. *Mol Cell Biol* **14**, 509-517 (1994).
230 Matsubayashi, Y., Fukuda, M. & Nishida, E. Evidence for existence of a nuclear pore complex-mediated, cytosol-independent pathway of nuclear translocation of ERK MAP kinase in permeabilized cells. *J Biol Chem* **276**, 41755-41760, doi:10.1074/jbc.M106012200 (2001).
231 Nakielny, S., Cohen, P., Wu, J. & Sturgill, T. MAP kinase activator from insulin-stimulated skeletal muscle is a protein threonine/tyrosine kinase. *EMBO J* **11**, 2123-2129 (1992).
232 Crews, C. M., Alessandrini, A. & Erikson, R. L. The primary structure of MEK, a protein kinase that phosphorylates the ERK gene product. *Science* **258**, 478-480 (1992).
233 Frodin, M. & Gammeltoft, S. Role and regulation of 90 kDa ribosomal S6 kinase (RSK) in signal transduction. *Mol Cell Endocrinol* **151**, 65-77 (1999).
234 Jensen, C. J. *et al.* 90-kDa ribosomal S6 kinase is phosphorylated and activated by 3-phosphoinositide-dependent protein kinase-1. *J Biol Chem* **274**, 27168-27176 (1999).
235 Chrestensen, C. A. & Sturgill, T. W. Characterization of the p90 ribosomal S6 kinase 2 carboxyl-terminal domain as a protein kinase. *J Biol Chem* **277**, 27733-27741, doi:10.1074/jbc.M202663200 (2002).
236 Frodin, M., Jensen, C. J., Merienne, K. & Gammeltoft, S. A phosphoserine-regulated docking site in the protein kinase RSK2 that recruits and activates PDK1. *EMBO J* **19**, 2924-2934, doi:10.1093/emboj/19.12.2924 (2000).
237 Poteet-Smith, C. E., Smith, J. A., Lannigan, D. A., Freed, T. A. & Sturgill, T. W. Generation of constitutively active p90 ribosomal S6 kinase in vivo. Implications for the mitogen-activated protein kinase-activated protein kinase family. *J Biol Chem* **274**, 22135-22138 (1999).
238 Dong, C., Waters, S. B., Holt, K. H. & Pessin, J. E. SOS phosphorylation and disassociation of the Grb2-SOS complex by the ERK and JNK signaling pathways. *J Biol Chem* **271**, 6328-6332 (1996).
239 Bunda, S. *et al.* Inhibition of SHP2-mediated dephosphorylation of Ras suppresses oncogenesis. *Nat Commun* **6**, 8859, doi:10.1038/ncomms9859 (2015).
240 Coronella-Wood, J., Terrand, J., Sun, H. & Chen, Q. M. c-Fos phosphorylation induced by H2O2 prevents proteasomal degradation of c-Fos in cardiomyocytes. *J Biol Chem* **279**, 33567-33574, doi:10.1074/jbc.M404013200 (2004).

241 Zhang, H. H., Lipovsky, A. I., Dibble, C. C., Sahin, M. & Manning, B. D. S6K1 regulates GSK3 under conditions of mTOR-dependent feedback inhibition of Akt. *Mol Cell* **24**, 185-197, doi:10.1016/j.molcel.2006.09.019 (2006).
242 Sutherland, C., Leighton, I. A. & Cohen, P. Inactivation of glycogen synthase kinase-3 beta by phosphorylation: new kinase connections in insulin and growth-factor signalling. *Biochem J* **296 (Pt 1)**, 15-19 (1993).
243 Cohen, P. & Frame, S. The renaissance of GSK3. *Nat Rev Mol Cell Biol* **2**, 769-776, doi:10.1038/35096075 (2001).
244 Jope, R. S. & Johnson, G. V. The glamour and gloom of glycogen synthase kinase-3. *Trends Biochem Sci* **29**, 95-102, doi:10.1016/j.tibs.2003.12.004 (2004).
245 Patel, S., Doble, B. & Woodgett, J. R. Glycogen synthase kinase-3 in insulin and Wnt signalling: a double-edged sword? *Biochem Soc Trans* **32**, 803-808, doi:10.1042/BST0320803 (2004).
246 Kobayashi, T. & Cohen, P. Activation of serum- and glucocorticoid-regulated protein kinase by agonists that activate phosphatidylinositide 3-kinase is mediated by 3-phosphoinositide-dependent protein kinase-1 (PDK1) and PDK2. *Biochem J* **339 (Pt 2)**, 319-328 (1999).
247 Biondi, R. M., Kieloch, A., Currie, R. A., Deak, M. & Alessi, D. R. The PIF-binding pocket in PDK1 is essential for activation of S6K and SGK, but not PKB. *EMBO J* **20**, 4380-4390, doi:10.1093/emboj/20.16.4380 (2001).
248 Park, J. *et al.* Serum and glucocorticoid-inducible kinase (SGK) is a target of the PI 3-kinase-stimulated signaling pathway. *EMBO J* **18**, 3024-3033, doi:10.1093/emboj/18.11.3024 (1999).
249 Garcia-Martinez, J. M. & Alessi, D. R. mTOR complex 2 (mTORC2) controls hydrophobic motif phosphorylation and activation of serum- and glucocorticoid-induced protein kinase 1 (SGK1). *Biochem J* **416**, 375-385, doi:10.1042/BJ20081668 (2008).
250 Yan, L., Mieulet, V. & Lamb, R. F. mTORC2 is the hydrophobic motif kinase for SGK1. *Biochem J* **416**, e19-21, doi:10.1042/BJ20082202 (2008).
251 Sarbassov, D. D. *et al.* Rictor, a novel binding partner of mTOR, defines a rapamycin-insensitive and raptor-independent pathway that regulates the cytoskeleton. *Curr Biol* **14**, 1296-1302, doi:10.1016/j.cub.2004.06.054 (2004).
252 Guertin, D. A. *et al.* Ablation in mice of the mTORC components raptor, rictor, or mLST8 reveals that mTORC2 is required for signaling to Akt-FOXO and PKCalpha, but not S6K1. *Dev Cell* **11**, 859-871, doi:10.1016/j.devcel.2006.10.007 (2006).
253 Ikenoue, T., Inoki, K., Yang, Q., Zhou, X. & Guan, K. L. Essential function of TORC2 in PKC and Akt turn motif phosphorylation, maturation and signalling. *EMBO J* **27**, 1919-1931, doi:10.1038/emboj.2008.119 (2008).
254 Dutil, E. M., Toker, A. & Newton, A. C. Regulation of conventional protein kinase C isozymes by phosphoinositide-dependent kinase 1 (PDK-1). *Curr Biol* **8**, 1366-1375 (1998).
255 Sonnenburg, E. D., Gao, T. & Newton, A. C. The phosphoinositide-dependent kinase, PDK-1, phosphorylates conventional protein kinase C isozymes by a mechanism that is independent of phosphoinositide 3-kinase. *J Biol Chem* **276**, 45289-45297, doi:10.1074/jbc.M107416200 (2001).
256 Toyofuku, T., Curotto Kurzydlowski, K., Narayanan, N. & MacLennan, D. H. Identification of Ser38 as the site in cardiac sarcoplasmic reticulum Ca(2+)-ATPase that is phosphorylated by Ca2+/calmodulin-dependent protein kinase. *J Biol Chem* **269**, 26492-26496 (1994).
257 DeSouza, N. *et al.* Protein kinase A and two phosphatases are components of the inositol 1,4,5-trisphosphate receptor macromolecular signaling complex. *J Biol Chem* **277**, 39397-39400, doi:10.1074/jbc.M207059200 (2002).

258 Zhang, B. & Zheng, Y. Regulation of RhoA GTP hydrolysis by the GTPase-activating proteins p190, p50RhoGAP, Bcr, and 3BP-1. *Biochemistry* **37**, 5249-5257, doi:10.1021/bi9718447 (1998).
259 Vogt, S., Grosse, R., Schultz, G. & Offermanns, S. Receptor-dependent RhoA activation in G12/G13-deficient cells: genetic evidence for an involvement of Gq/G11. *J Biol Chem* **278**, 28743-28749, doi:10.1074/jbc.M304570200 (2003).
260 Johnston, C. A. & Watts, V. J. Sensitization of adenylate cyclase: a general mechanism of neuroadaptation to persistent activation of Galpha(i/o)-coupled receptors? *Life Sci* **73**, 2913-2925 (2003).
261 Beebe, S. J. The cAMP-dependent protein kinases and cAMP signal transduction. *Semin Cancer Biol* **5**, 285-294 (1994).
262 Banno, Y., Asano, T. & Nozawa, Y. Proteolytic modification of membrane-associated phospholipase C-beta by mu-calpain enhances its activation by G-protein beta gamma subunits in human platelets. *FEBS Lett* **340**, 185-188 (1994).
263 Yue, C., Ku, C. Y., Liu, M., Simon, M. I. & Sanborn, B. M. Molecular mechanism of the inhibition of phospholipase C beta 3 by protein kinase C. *J Biol Chem* **275**, 30220-30225, doi:10.1074/jbc.M004276200 (2000).
264 Cardone, M. H. *et al.* Regulation of cell death protease caspase-9 by phosphorylation. *Science* **282**, 1318-1321 (1998).
265 Djouder, N. *et al.* S6K1-mediated disassembly of mitochondrial URI/PP1gamma complexes activates a negative feedback program that counters S6K1 survival signaling. *Mol Cell* **28**, 28-40, doi:10.1016/j.molcel.2007.08.010 (2007).
266 Harada, H., Andersen, J. S., Mann, M., Terada, N. & Korsmeyer, S. J. p70S6 kinase signals cell survival as well as growth, inactivating the pro-apoptotic molecule BAD. *Proc Natl Acad Sci U S A* **98**, 9666-9670, doi:10.1073/pnas.171301998 (2001).
267 Gottlieb, T. M., Leal, J. F., Seger, R., Taya, Y. & Oren, M. Cross-talk between Akt, p53 and Mdm2: possible implications for the regulation of apoptosis. *Oncogene* **21**, 1299-1303, doi:10.1038/sj.onc.1205181 (2002).
268 Moritz, A., De Graan, P. N., Gispen, W. H. & Wirtz, K. W. Phosphatidic acid is a specific activator of phosphatidylinositol-4-phosphate kinase. *J Biol Chem* **267**, 7207-7210 (1992).
269 Bozulic, L. & Hemmings, B. A. PIKKing on PKB: regulation of PKB activity by phosphorylation. *Curr Opin Cell Biol* **21**, 256-261, doi:10.1016/j.ceb.2009.02.002 (2009).
270 Manning, B. D. & Toker, A. AKT/PKB Signaling: Navigating the Network. *Cell* **169**, 381-405, doi:10.1016/j.cell.2017.04.001 (2017).
271 Facchinetti, V. *et al.* The mammalian target of rapamycin complex 2 controls folding and stability of Akt and protein kinase C. *EMBO J* **27**, 1932-1943, doi:10.1038/emboj.2008.120 (2008).
272 Scheid, M. P. & Woodgett, J. R. Unravelling the activation mechanisms of protein kinase B/Akt. *FEBS Lett* **546**, 108-112 (2003).
273 Oudit, G. Y. *et al.* The role of phosphoinositide-3 kinase and PTEN in cardiovascular physiology and disease. *J Mol Cell Cardiol* **37**, 449-471, doi:10.1016/j.yjmcc.2004.05.015 (2004).
274 Vanhaesebroeck, B. *et al.* Synthesis and function of 3-phosphorylated inositol lipids. *Annu Rev Biochem* **70**, 535-602, doi:10.1146/annurev.biochem.70.1.535 (2001).
275 Tolias, K. F. & Cantley, L. C. Pathways for phosphoinositide synthesis. *Chem Phys Lipids* **98**, 69-77 (1999).
276 Stephens, L. R., Jackson, T. R. & Hawkins, P. T. Agonist-stimulated synthesis of phosphatidylinositol(3,4,5)-trisphosphate: a new intracellular signalling system? *Biochim Biophys Acta* **1179**, 27-75 (1993).

277 Miyake, S., Lupher, M. L., Jr., Druker, B. & Band, H. The tyrosine kinase regulator Cbl enhances the ubiquitination and degradation of the platelet-derived growth factor receptor alpha. *Proc Natl Acad Sci U S A* **95**, 7927-7932 (1998).
278 Bonita, D. P., Miyake, S., Lupher, M. L., Jr., Langdon, W. Y. & Band, H. Phosphotyrosine binding domain-dependent upregulation of the platelet-derived growth factor receptor alpha signaling cascade by transforming mutants of Cbl: implications for Cbl's function and oncogenicity. *Mol Cell Biol* **17**, 4597-4610 (1997).
279 Sato, S., Fujita, N. & Tsuruo, T. Modulation of Akt kinase activity by binding to Hsp90. *Proc Natl Acad Sci U S A* **97**, 10832-10837, doi:10.1073/pnas.170276797 (2000).
280 Acosta-Jaquez, H. A. *et al.* Site-specific mTOR phosphorylation promotes mTORC1-mediated signaling and cell growth. *Mol Cell Biol* **29**, 4308-4324, doi:10.1128/MCB.01665-08 (2009).
281 Peterson, R. T., Beal, P. A., Comb, M. J. & Schreiber, S. L. FKBP12-rapamycin-associated protein (FRAP) autophosphorylates at serine 2481 under translationally repressive conditions. *J Biol Chem* **275**, 7416-7423 (2000).
282 Cheng, S. W., Fryer, L. G., Carling, D. & Shepherd, P. R. Thr2446 is a novel mammalian target of rapamycin (mTOR) phosphorylation site regulated by nutrient status. *J Biol Chem* **279**, 15719-15722, doi:10.1074/jbc.C300534200 (2004).
283 Chiang, G. G. & Abraham, R. T. Phosphorylation of mammalian target of rapamycin (mTOR) at Ser-2448 is mediated by p70S6 kinase. *J Biol Chem* **280**, 25485-25490, doi:10.1074/jbc.M501707200 (2005).
284 Holz, M. K. & Blenis, J. Identification of S6 kinase 1 as a novel mammalian target of rapamycin (mTOR)-phosphorylating kinase. *J Biol Chem* **280**, 26089-26093, doi:10.1074/jbc.M504045200 (2005).
285 Hara, K. *et al.* Raptor, a binding partner of target of rapamycin (TOR), mediates TOR action. *Cell* **110**, 177-189 (2002).
286 Kim, D. H. *et al.* mTOR interacts with raptor to form a nutrient-sensitive complex that signals to the cell growth machinery. *Cell* **110**, 163-175 (2002).
287 Loewith, R. *et al.* Two TOR complexes, only one of which is rapamycin sensitive, have distinct roles in cell growth control. *Mol Cell* **10**, 457-468 (2002).
288 Velasquez, C. *et al.* Mitotic protein kinase CDK1 phosphorylation of mRNA translation regulator 4E-BP1 Ser83 may contribute to cell transformation. *Proc Natl Acad Sci U S A* **113**, 8466-8471, doi:10.1073/pnas.1607768113 (2016).
289 Phin, S., Kupferwasser, D., Lam, J. & Lee-Fruman, K. K. Mutational analysis of ribosomal S6 kinase 2 shows differential regulation of its kinase activity from that of ribosomal S6 kinase 1. *Biochem J* **373**, 583-591, doi:10.1042/BJ20021794 (2003).
290 Minami, T. *et al.* Distinct regulatory mechanism for p70 S6 kinase beta from that for p70 S6 kinase alpha. *Genes Cells* **6**, 1003-1015 (2001).
291 Martin, K. A., Schalm, S. S., Romanelli, A., Keon, K. L. & Blenis, J. Ribosomal S6 kinase 2 inhibition by a potent C-terminal repressor domain is relieved by mitogen-activated protein-extracellular signal-regulated kinase kinase-regulated phosphorylation. *J Biol Chem* **276**, 7892-7898, doi:10.1074/jbc.M009972200 (2001).
292 Dibble, C. C., Asara, J. M. & Manning, B. D. Characterization of Rictor phosphorylation sites reveals direct regulation of mTOR complex 2 by S6K1. *Mol Cell Biol* **29**, 5657-5670, doi:10.1128/MCB.00735-09 (2009).
293 Panasyuk, G., Nemazanyy, I., Filonenko, V. & Gout, I. Ribosomal protein S6 kinase 1 interacts with and is ubiquitinated by ubiquitin ligase ROC1. *Biochem Biophys Res Commun* **369**, 339-343, doi:10.1016/j.bbrc.2008.02.016 (2008).
294 Peterson, R. T., Desai, B. N., Hardwick, J. S. & Schreiber, S. L. Protein phosphatase 2A interacts with the 70-kDa S6 kinase and is activated by inhibition of FKBP12-rapamycinassociated protein. *Proc Natl Acad Sci U S A* **96**, 4438-4442 (1999).

295 Yang, Q., Inoki, K., Kim, E. & Guan, K. L. TSC1/TSC2 and Rheb have different effects on TORC1 and TORC2 activity. *Proc Natl Acad Sci U S A* **103**, 6811-6816, doi:10.1073/pnas.0602282103 (2006).
296 Roux, P. P. *et al.* RAS/ERK signaling promotes site-specific ribosomal protein S6 phosphorylation via RSK and stimulates cap-dependent translation. *J Biol Chem* **282**, 14056-14064, doi:10.1074/jbc.M700906200 (2007).
297 Pende, M. *et al.* S6K1(-/-)/S6K2(-/-) mice exhibit perinatal lethality and rapamycin-sensitive 5'-terminal oligopyrimidine mRNA translation and reveal a mitogen-activated protein kinase-dependent S6 kinase pathway. *Mol Cell Biol* **24**, 3112-3124 (2004).
298 Dorrello, N. V. *et al.* S6K1- and betaTRCP-mediated degradation of PDCD4 promotes protein translation and cell growth. *Science* **314**, 467-471, doi:10.1126/science.1130276 (2006).
299 Pyronnet, S. *et al.* Human eukaryotic translation initiation factor 4G (eIF4G) recruits mnk1 to phosphorylate eIF4E. *EMBO J* **18**, 270-279, doi:10.1093/emboj/18.1.270 (1999).
300 Ueda, T., Watanabe-Fukunaga, R., Fukuyama, H., Nagata, S. & Fukunaga, R. Mnk2 and Mnk1 are essential for constitutive and inducible phosphorylation of eukaryotic initiation factor 4E but not for cell growth or development. *Mol Cell Biol* **24**, 6539-6549, doi:10.1128/MCB.24.15.6539-6549.2004 (2004).
301 Lamphear, B. J. & Panniers, R. Cap binding protein complex that restores protein synthesis in heat-shocked Ehrlich cell lysates contains highly phosphorylated eIF-4E. *J Biol Chem* **265**, 5333-5336 (1990).
302 Joshi-Barve, S., Rychlik, W. & Rhoads, R. E. Alteration of the major phosphorylation site of eukaryotic protein synthesis initiation factor 4E prevents its association with the 48 S initiation complex. *J Biol Chem* **265**, 2979-2983 (1990).
303 Kleijn, M., Scheper, G. C., Voorma, H. O. & Thomas, A. A. Regulation of translation initiation factors by signal transduction. *Eur J Biochem* **253**, 531-544 (1998).
304 Saghir, A. N., Tuxworth, W. J., Jr., Hagedorn, C. H. & McDermott, P. J. Modifications of eukaryotic initiation factor 4F (eIF4F) in adult cardiocytes by adenoviral gene transfer: differential effects on eIF4F activity and total protein synthesis rates. *Biochem J* **356**, 557-566 (2001).
305 McKendrick, L., Morley, S. J., Pain, V. M., Jagus, R. & Joshi, B. Phosphorylation of eukaryotic initiation factor 4E (eIF4E) at Ser209 is not required for protein synthesis in vitro and in vivo. *Eur J Biochem* **268**, 5375-5385 (2001).
306 Scheper, G. C. *et al.* Phosphorylation of eukaryotic initiation factor 4E markedly reduces its affinity for capped mRNA. *J Biol Chem* **277**, 3303-3309, doi:10.1074/jbc.M103607200 (2002).
307 Wendel, H. G. *et al.* Dissecting eIF4E action in tumorigenesis. *Genes Dev* **21**, 3232-3237, doi:10.1101/gad.1604407 (2007).
308 Browne, G. J., Finn, S. G. & Proud, C. G. Stimulation of the AMP-activated protein kinase leads to activation of eukaryotic elongation factor 2 kinase and to its phosphorylation at a novel site, serine 398. *J Biol Chem* **279**, 12220-12231, doi:10.1074/jbc.M309773200 (2004).
309 Knebel, A., Morrice, N. & Cohen, P. A novel method to identify protein kinase substrates: eEF2 kinase is phosphorylated and inhibited by SAPK4/p38delta. *EMBO J* **20**, 4360-4369, doi:10.1093/emboj/20.16.4360 (2001).
310 Diggle, T. A., Redpath, N. T., Heesom, K. J. & Denton, R. M. Regulation of protein-synthesis elongation-factor-2 kinase by cAMP in adipocytes. *Biochem J* **336 (Pt 3)**, 525-529 (1998).
311 Wang, X. *et al.* Regulation of elongation factor 2 kinase by p90(RSK1) and p70 S6 kinase. *EMBO J* **20**, 4370-4379, doi:10.1093/emboj/20.16.4370 (2001).

312 Guillot, D. *et al.* GTP binding to elongation factor eEF-2 unmasks a tryptophan residue required for biological activity. *J Biol Chem* **268**, 20911-20916 (1993).
313 Le Sourd, F. *et al.* eEF1B: At the dawn of the 21st century. *Biochim Biophys Acta* **1759**, 13-31, doi:10.1016/j.bbaexp.2006.02.003 (2006).
314 Venema, R. C., Peters, H. I. & Traugh, J. A. Phosphorylation of elongation factor 1 (EF-1) and valyl-tRNA synthetase by protein kinase C and stimulation of EF-1 activity. *J Biol Chem* **266**, 12574-12580 (1991).
315 Kawaguchi, Y. & Kato, K. Protein kinases conserved in herpesviruses potentially share a function mimicking the cellular protein kinase cdc2. *Rev Med Virol* **13**, 331-340, doi:10.1002/rmv.402 (2003).
316 Gyenis, L., Duncan, J. S., Turowec, J. P., Bretner, M. & Litchfield, D. W. Unbiased functional proteomics strategy for protein kinase inhibitor validation and identification of bona fide protein kinase substrates: application to identification of EEF1D as a substrate for CK2. *J Proteome Res* **10**, 4887-4901, doi:10.1021/pr2008994 (2011).
317 Fan, Y. *et al.* Drosophila translational elongation factor-1gamma is modified in response to DOA kinase activity and is essential for cellular viability. *Genetics* **184**, 141-154, doi:10.1534/genetics.109.109553 (2010).
318 Mulner-Lorillon, O. *et al.* Phosphorylation of Xenopus elongation factor-1 gamma by cdc2 protein kinase: identification of the phosphorylation site. *Exp Cell Res* **202**, 549-551 (1992).
319 Janssen, G. M., Maessen, G. D., Amons, R. & Moller, W. Phosphorylation of elongation factor 1 beta by an endogenous kinase affects its catalytic nucleotide exchange activity. *J Biol Chem* **263**, 11063-11066 (1988).
320 Chen, C. J. & Traugh, J. A. Expression of recombinant elongation factor 1 beta from rabbit in Escherichia coli. Phosphorylation by casein kinase II. *Biochim Biophys Acta* **1264**, 303-311 (1995).
321 Shahbazian, D. *et al.* The mTOR/PI3K and MAPK pathways converge on eIF4B to control its phosphorylation and activity. *EMBO J* **25**, 2781-2791, doi:10.1038/sj.emboj.7601166 (2006).
322 Cen, B. *et al.* The Pim-1 protein kinase is an important regulator of MET receptor tyrosine kinase levels and signaling. *Mol Cell Biol* **34**, 2517-2532, doi:10.1128/MCB.00147-14 (2014).
323 Raught, B. *et al.* Phosphorylation of eucaryotic translation initiation factor 4B Ser422 is modulated by S6 kinases. *EMBO J* **23**, 1761-1769, doi:10.1038/sj.emboj.7600193 (2004).
324 Raught, B. *et al.* Serum-stimulated, rapamycin-sensitive phosphorylation sites in the eukaryotic translation initiation factor 4GI. *EMBO J* **19**, 434-444, doi:10.1093/emboj/19.3.434 (2000).
325 DuRose, J. B., Scheuner, D., Kaufman, R. J., Rothblum, L. I. & Niwa, M. Phosphorylation of eukaryotic translation initiation factor 2alpha coordinates rRNA transcription and translation inhibition during endoplasmic reticulum stress. *Mol Cell Biol* **29**, 4295-4307, doi:10.1128/MCB.00260-09 (2009).
326 Garcia, M. A., Meurs, E. F. & Esteban, M. The dsRNA protein kinase PKR: virus and cell control. *Biochimie* **89**, 799-811, doi:10.1016/j.biochi.2007.03.001 (2007).
327 Harding, H. P., Zhang, Y. & Ron, D. Protein translation and folding are coupled by an endoplasmic-reticulum-resident kinase. *Nature* **397**, 271-274, doi:10.1038/16729 (1999).
328 Kubota, H., Obata, T., Ota, K., Sasaki, T. & Ito, T. Rapamycin-induced translational derepression of GCN4 mRNA involves a novel mechanism for activation of the eIF2 alpha kinase GCN2. *J Biol Chem* **278**, 20457-20460, doi:10.1074/jbc.C300133200 (2003).

329 Lu, L., Han, A. P. & Chen, J. J. Translation initiation control by heme-regulated eukaryotic initiation factor 2alpha kinase in erythroid cells under cytoplasmic stresses. *Mol Cell Biol* **21**, 7971-7980, doi:10.1128/MCB.21.23.7971-7980.2001 (2001).
330 Zhang, P. *et al.* The GCN2 eIF2alpha kinase is required for adaptation to amino acid deprivation in mice. *Mol Cell Biol* **22**, 6681-6688 (2002).
331 Colthurst, D. R., Campbell, D. G. & Proud, C. G. Structure and regulation of eukaryotic initiation factor eIF-2. Sequence of the site in the alpha subunit phosphorylated by the haem-controlled repressor and by the double-stranded RNA-activated inhibitor. *Eur J Biochem* **166**, 357-363 (1987).
332 Wang, X. *et al.* Eukaryotic initiation factor 2B: identification of multiple phosphorylation sites in the epsilon-subunit and their functions in vivo. *EMBO J* **20**, 4349-4359, doi:10.1093/emboj/20.16.4349 (2001).
333 Welsh, G. I., Miller, C. M., Loughlin, A. J., Price, N. T. & Proud, C. G. Regulation of eukaryotic initiation factor eIF2B: glycogen synthase kinase-3 phosphorylates a conserved serine which undergoes dephosphorylation in response to insulin. *FEBS Lett* **421**, 125-130 (1998).
334 Welsh, G. I. & Proud, C. G. Glycogen synthase kinase-3 is rapidly inactivated in response to insulin and phosphorylates eukaryotic initiation factor eIF-2B. *Biochem J* **294 (Pt 3)**, 625-629 (1993).
335 Woods, Y. L. *et al.* The kinase DYRK phosphorylates protein-synthesis initiation factor eIF2Bepsilon at Ser539 and the microtubule-associated protein tau at Thr212: potential role for DYRK as a glycogen synthase kinase 3-priming kinase. *Biochem J* **355**, 609-615 (2001).
336 Boesen, T., Mohammad, S. S., Pavitt, G. D. & Andersen, G. R. Structure of the catalytic fragment of translation initiation factor 2B and identification of a critically important catalytic residue. *J Biol Chem* **279**, 10584-10592, doi:10.1074/jbc.M311055200 (2004).
337 Gingras, A. C. *et al.* Hierarchical phosphorylation of the translation inhibitor 4E-BP1. *Genes Dev* **15**, 2852-2864, doi:10.1101/gad.912401 (2001).
338 Beretta, L., Gingras, A. C., Svitkin, Y. V., Hall, M. N. & Sonenberg, N. Rapamycin blocks the phosphorylation of 4E-BP1 and inhibits cap-dependent initiation of translation. *EMBO J* **15**, 658-664 (1996).
339 Hara, K. *et al.* Regulation of eIF-4E BP1 phosphorylation by mTOR. *J Biol Chem* **272**, 26457-26463 (1997).
340 Gingras, A. C., Kennedy, S. G., O'Leary, M. A., Sonenberg, N. & Hay, N. 4E-BP1, a repressor of mRNA translation, is phosphorylated and inactivated by the Akt(PKB) signaling pathway. *Genes Dev* **12**, 502-513 (1998).
341 Alessi, D. R., Kozlowski, M. T., Weng, Q. P., Morrice, N. & Avruch, J. 3-Phosphoinositide-dependent protein kinase 1 (PDK1) phosphorylates and activates the p70 S6 kinase in vivo and in vitro. *Curr Biol* **8**, 69-81 (1998).
342 Pullen, N. *et al.* Phosphorylation and activation of p70s6k by PDK1. *Science* **279**, 707-710 (1998).
343 Frodin, M. *et al.* A phosphoserine/threonine-binding pocket in AGC kinases and PDK1 mediates activation by hydrophobic motif phosphorylation. *EMBO J* **21**, 5396-5407 (2002).
344 Dennis, P. B., Pullen, N., Pearson, R. B., Kozma, S. C. & Thomas, G. Phosphorylation sites in the autoinhibitory domain participate in p70(s6k) activation loop phosphorylation. *J Biol Chem* **273**, 14845-14852 (1998).
345 Ferrari, S., Bannwarth, W., Morley, S. J., Totty, N. F. & Thomas, G. Activation of p70s6k is associated with phosphorylation of four clustered sites displaying Ser/Thr-Pro motifs. *Proc Natl Acad Sci U S A* **89**, 7282-7286 (1992).

346 Isotani, S. *et al.* Immunopurified mammalian target of rapamycin phosphorylates and activates p70 S6 kinase alpha in vitro. *J Biol Chem* **274**, 34493-34498 (1999).
347 Moser, B. A. *et al.* Dual requirement for a newly identified phosphorylation site in p70s6k. *Mol Cell Biol* **17**, 5648-5655 (1997).
348 Saitoh, M. *et al.* Regulation of an activated S6 kinase 1 variant reveals a novel mammalian target of rapamycin phosphorylation site. *J Biol Chem* **277**, 20104-20112, doi:10.1074/jbc.M201745200 (2002).
349 Pearson, R. B. *et al.* The principal target of rapamycin-induced p70s6k inactivation is a novel phosphorylation site within a conserved hydrophobic domain. *EMBO J* **14**, 5279-5287 (1995).
350 Roux, P. P., Ballif, B. A., Anjum, R., Gygi, S. P. & Blenis, J. Tumor-promoting phorbol esters and activated Ras inactivate the tuberous sclerosis tumor suppressor complex via p90 ribosomal S6 kinase. *Proc Natl Acad Sci U S A* **101**, 13489-13494, doi:10.1073/pnas.0405659101 (2004).
351 Inoki, K., Zhu, T. & Guan, K. L. TSC2 mediates cellular energy response to control cell growth and survival. *Cell* **115**, 577-590 (2003).
352 Minden, A. *et al.* c-Jun N-terminal phosphorylation correlates with activation of the JNK subgroup but not the ERK subgroup of mitogen-activated protein kinases. *Mol Cell Biol* **14**, 6683-6688 (1994).
353 Hibi, M., Lin, A., Smeal, T., Minden, A. & Karin, M. Identification of an oncoprotein- and UV-responsive protein kinase that binds and potentiates the c-Jun activation domain. *Genes Dev* **7**, 2135-2148 (1993).
354 Smeal, T., Binetruy, B., Mercola, D. A., Birrer, M. & Karin, M. Oncogenic and transcriptional cooperation with Ha-Ras requires phosphorylation of c-Jun on serines 63 and 73. *Nature* **354**, 494-496, doi:10.1038/354494a0 (1991).
355 Hammond, S. M. *et al.* Characterization of two alternately spliced forms of phospholipase D1. Activation of the purified enzymes by phosphatidylinositol 4,5-bisphosphate, ADP-ribosylation factor, and Rho family monomeric GTP-binding proteins and protein kinase C-alpha. *J Biol Chem* **272**, 3860-3868 (1997).
356 Colley, W. C. *et al.* Phospholipase D2, a distinct phospholipase D isoform with novel regulatory properties that provokes cytoskeletal reorganization. *Curr Biol* **7**, 191-201 (1997).
357 Exton, J. H. Regulation of phospholipase D. *FEBS Lett* **531**, 58-61 (2002).
358 Caloca, M. J., Wang, H. & Kazanietz, M. G. Characterization of the Rac-GAP (Rac-GTPase-activating protein) activity of beta2-chimaerin, a 'non-protein kinase C' phorbol ester receptor. *Biochem J* **375**, 313-321, doi:10.1042/BJ20030727 (2003).
359 Menna, P. L. *et al.* Inhibition of aggressiveness of metastatic mouse mammary carcinoma cells by the beta2-chimaerin GAP domain. *Cancer Res* **63**, 2284-2291 (2003).
360 Jiang, X. & Sorkin, A. Coordinated traffic of Grb2 and Ras during epidermal growth factor receptor endocytosis visualized in living cells. *Mol Biol Cell* **13**, 1522-1535, doi:10.1091/mbc.01-11-0552 (2002).
361 Shields, J. M., Pruitt, K., McFall, A., Shaub, A. & Der, C. J. Understanding Ras: 'it ain't over 'til it's over'. *Trends Cell Biol* **10**, 147-154 (2000).
362 Moodie, S. A., Willumsen, B. M., Weber, M. J. & Wolfman, A. Complexes of Ras.GTP with Raf-1 and mitogen-activated protein kinase kinase. *Science* **260**, 1658-1661 (1993).
363 Carey, K. D., Watson, R. T., Pessin, J. E. & Stork, P. J. The requirement of specific membrane domains for Raf-1 phosphorylation and activation. *J Biol Chem* **278**, 3185-3196, doi:10.1074/jbc.M207014200 (2003).
364 Narayanan, N. & Xu, A. Phosphorylation and regulation of the Ca(2+)-pumping ATPase in cardiac sarcoplasmic reticulum by calcium/calmodulin-dependent protein kinase. *Basic Res Cardiol* **92 Suppl 1**, 25-35 (1997).

365 Ferguson, S. M. MEDICINE. Membrane traffic en route to cancer. *Science* **350**, 162-163, doi:10.1126/science.aad3575 (2015).
366 Wheeler, D. B., Zoncu, R., Root, D. E., Sabatini, D. M. & Sawyers, C. L. Identification of an oncogenic RAB protein. *Science* **350**, 211-217, doi:10.1126/science.aaa4903 (2015).
367 Yokote, K. *et al.* Direct interaction between Shc and the platelet-derived growth factor beta-receptor. *J Biol Chem* **269**, 15337-15343 (1994).
368 Rozakis-Adcock, M. *et al.* Association of the Shc and Grb2/Sem5 SH2-containing proteins is implicated in activation of the Ras pathway by tyrosine kinases. *Nature* **360**, 689-692, doi:10.1038/360689a0 (1992).
369 Kashishian, A., Kazlauskas, A. & Cooper, J. A. Phosphorylation sites in the PDGF receptor with different specificities for binding GAP and PI3 kinase in vivo. *EMBO J* **11**, 1373-1382 (1992).
370 Tu, Y., Li, F. & Wu, C. Nck-2, a novel Src homology2/3-containing adaptor protein that interacts with the LIM-only protein PINCH and components of growth factor receptor kinase-signaling pathways. *Mol Biol Cell* **9**, 3367-3382 (1998).
371 Nishimura, R. *et al.* Two signaling molecules share a phosphotyrosine-containing binding site in the platelet-derived growth factor receptor. *Mol Cell Biol* **13**, 6889-6896 (1993).
372 Kazlauskas, A., Feng, G. S., Pawson, T. & Valius, M. The 64-kDa protein that associates with the platelet-derived growth factor receptor beta subunit via Tyr-1009 is the SH2-containing phosphotyrosine phosphatase Syp. *Proc Natl Acad Sci U S A* **90**, 6939-6943 (1993).
373 Kawada, K. *et al.* Cell migration is regulated by platelet-derived growth factor receptor endocytosis. *Mol Cell Biol* **29**, 4508-4518, doi:10.1128/MCB.00015-09 (2009).
374 Arvidsson, A. K. *et al.* Tyr-716 in the platelet-derived growth factor beta-receptor kinase insert is involved in GRB2 binding and Ras activation. *Mol Cell Biol* **14**, 6715-6726 (1994).
375 Cote, J. F., Turner, C. E. & Tremblay, M. L. Intact LIM 3 and LIM 4 domains of paxillin are required for the association to a novel polyproline region (Pro 2) of protein-tyrosine phosphatase-PEST. *J Biol Chem* **274**, 20550-20560 (1999).
376 Mori, S. *et al.* Identification of two juxtamembrane autophosphorylation sites in the PDGF beta-receptor; involvement in the interaction with Src family tyrosine kinases. *EMBO J* **12**, 2257-2264 (1993).
377 Ronnstrand, L. *et al.* SHP-2 binds to Tyr763 and Tyr1009 in the PDGF beta-receptor and mediates PDGF-induced activation of the Ras/MAP kinase pathway and chemotaxis. *Oncogene* **18**, 3696-3702, doi:10.1038/sj.onc.1202705 (1999).
378 Han, J. *et al.* Role of substrates and products of PI 3-kinase in regulating activation of Rac-related guanosine triphosphatases by Vav. *Science* **279**, 558-560 (1998).
379 Izumi, H. *et al.* Mechanism for the transcriptional repression by c-Myc on PDGF beta-receptor. *J Cell Sci* **114**, 1533-1544 (2001).
380 Vanhoutte, P. *et al.* Glutamate induces phosphorylation of Elk-1 and CREB, along with c-fos activation, via an extracellular signal-regulated kinase-dependent pathway in brain slices. *Mol Cell Biol* **19**, 136-146 (1999).
381 el-Deiry, W. S. *et al.* WAF1, a potential mediator of p53 tumor suppression. *Cell* **75**, 817-825 (1993).
382 Reddi, A. L. *et al.* Binding of Cbl to a phospholipase Cgamma1-docking site on platelet-derived growth factor receptor beta provides a dual mechanism of negative regulation. *J Biol Chem* **282**, 29336-29347, doi:10.1074/jbc.M701797200 (2007).
383 Mori, S., Tanaka, K., Omura, S. & Saito, Y. Degradation process of ligand-stimulated platelet-derived growth factor beta-receptor involves ubiquitin-proteasome proteolytic pathway. *J Biol Chem* **270**, 29447-29452 (1995).

384 Chamberlain, M. D. *et al.* Deregulation of Rab5 and Rab4 proteins in p85R274A-expressing cells alters PDGFR trafficking. *Cell Signal* **22**, 1562-1575, doi:10.1016/j.cellsig.2010.05.025 (2010).
385 Dobbin, E. *et al.* Tel/PDGFRbeta induces stem cell differentiation via the Ras/ERK and STAT5 signaling pathways. *Exp Hematol* **37**, 111-121, doi:10.1016/j.exphem.2008.09.012 (2009).
386 Nakayama, A. *et al.* Ephrin-B2 controls PDGFRbeta internalization and signaling. *Genes Dev* **27**, 2576-2589, doi:10.1101/gad.224089.113 (2013).
387 Ungewickell, E. J. & Hinrichsen, L. Endocytosis: clathrin-mediated membrane budding. *Curr Opin Cell Biol* **19**, 417-425, doi:10.1016/j.ceb.2007.05.003 (2007).
388 Vanlandingham, P. A. & Ceresa, B. P. Rab7 regulates late endocytic trafficking downstream of multivesicular body biogenesis and cargo sequestration. *J Biol Chem* **284**, 12110-12124, doi:10.1074/jbc.M809277200 (2009).
389 Zerial, M. & McBride, H. Rab proteins as membrane organizers. *Nat Rev Mol Cell Biol* **2**, 107-117, doi:10.1038/35052055 (2001).
390 Zhu, J., Lin, F., Brown, D. A. & Clark, R. A. F. A fibronectin peptide redirects PDGF-BB/PDGFR complexes to macropinocytosis-like internalization and augments PDGF-BB survival signals. *J Invest Dermatol* **134**, 921-929, doi:10.1038/jid.2013.463 (2014).
