# Supplementary figures and images for: Chronic platelet-derived growth factor receptor signaling exerts control over initiation of protein translation in glioma
Source: Life Sci Alliance. 2018 Jun 19;1(3):e201800029. doi: 10.26508/lsa.201800029 (PMC6238596; doi:10.26508/lsa.201800029)

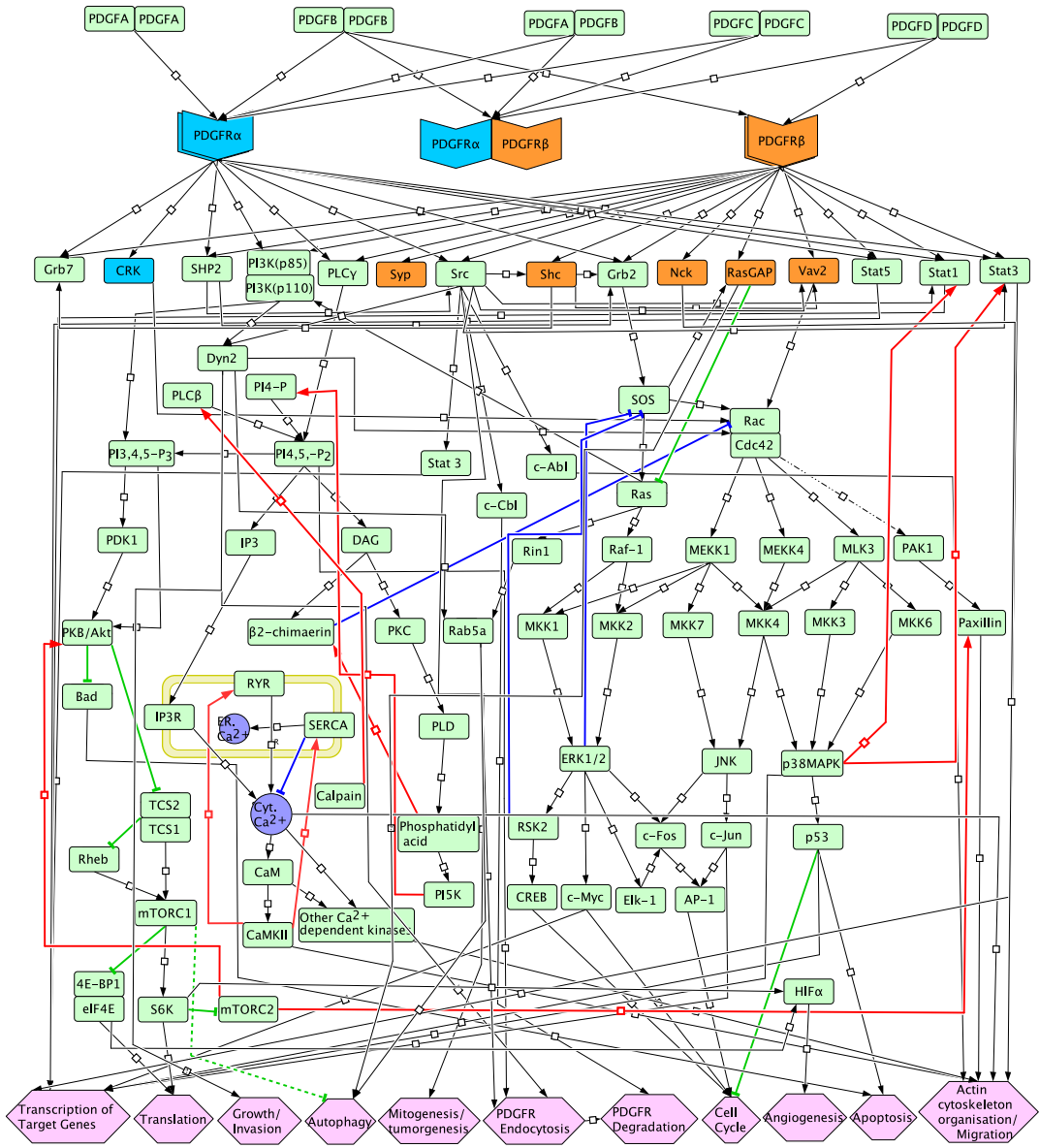

Supplement: Supplementary file 1 [file LSA-2018-00029_Preparation_of_compounds.zip › LSA-2018-00029_Data_file_1_(suppl_material)_PDF_format_file_for_PDGFR_signaling_activity_flow_Figure_Data_file_1_(suppl_material).pdf]

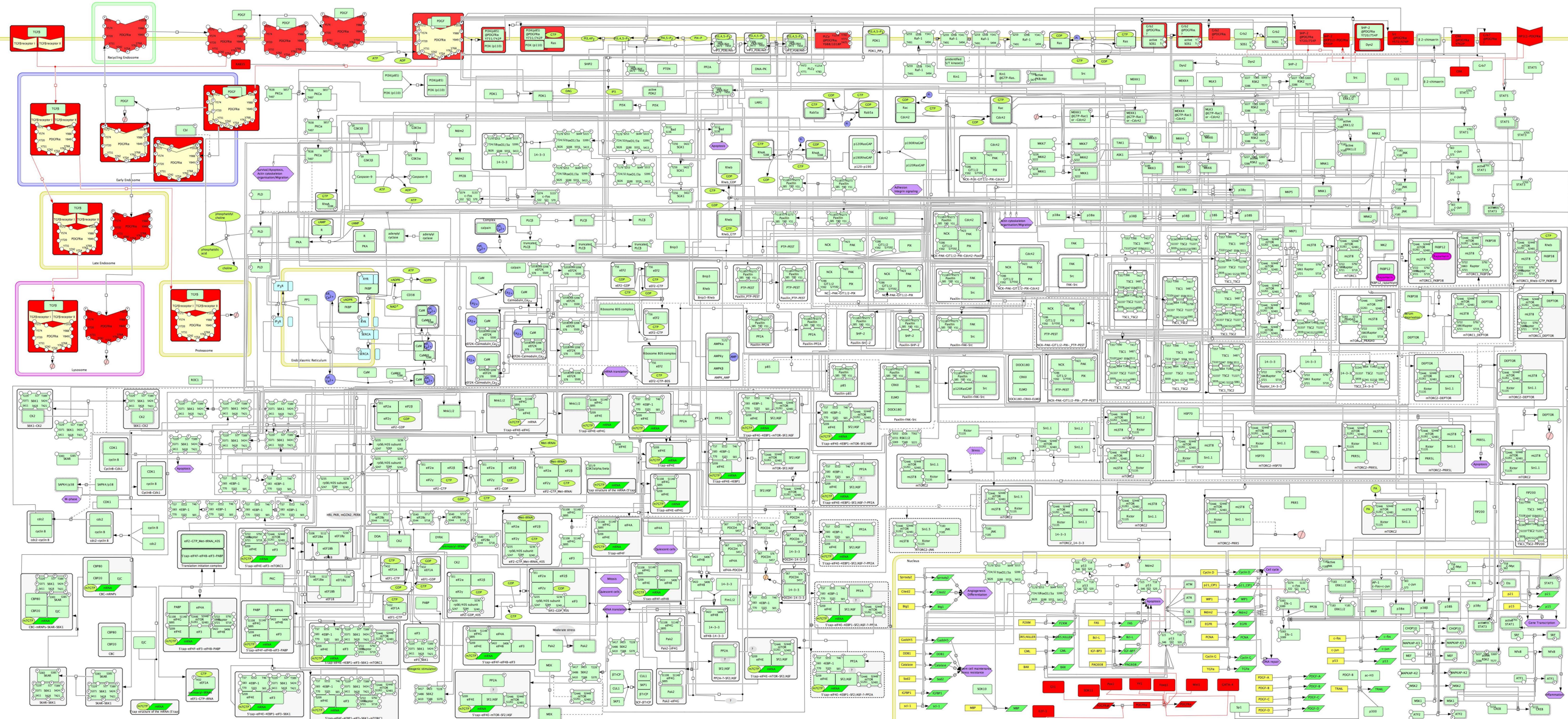

Supplement: Supplementary file 1 [file LSA-2018-00029_Preparation_of_compounds.zip › LSA-2018-00029_Data_file_2_(suppl_material)_PDF_format_file_for_PDGFRαα comprehensive_map_Figure_Data_file_2_(suppl_material).pdf]

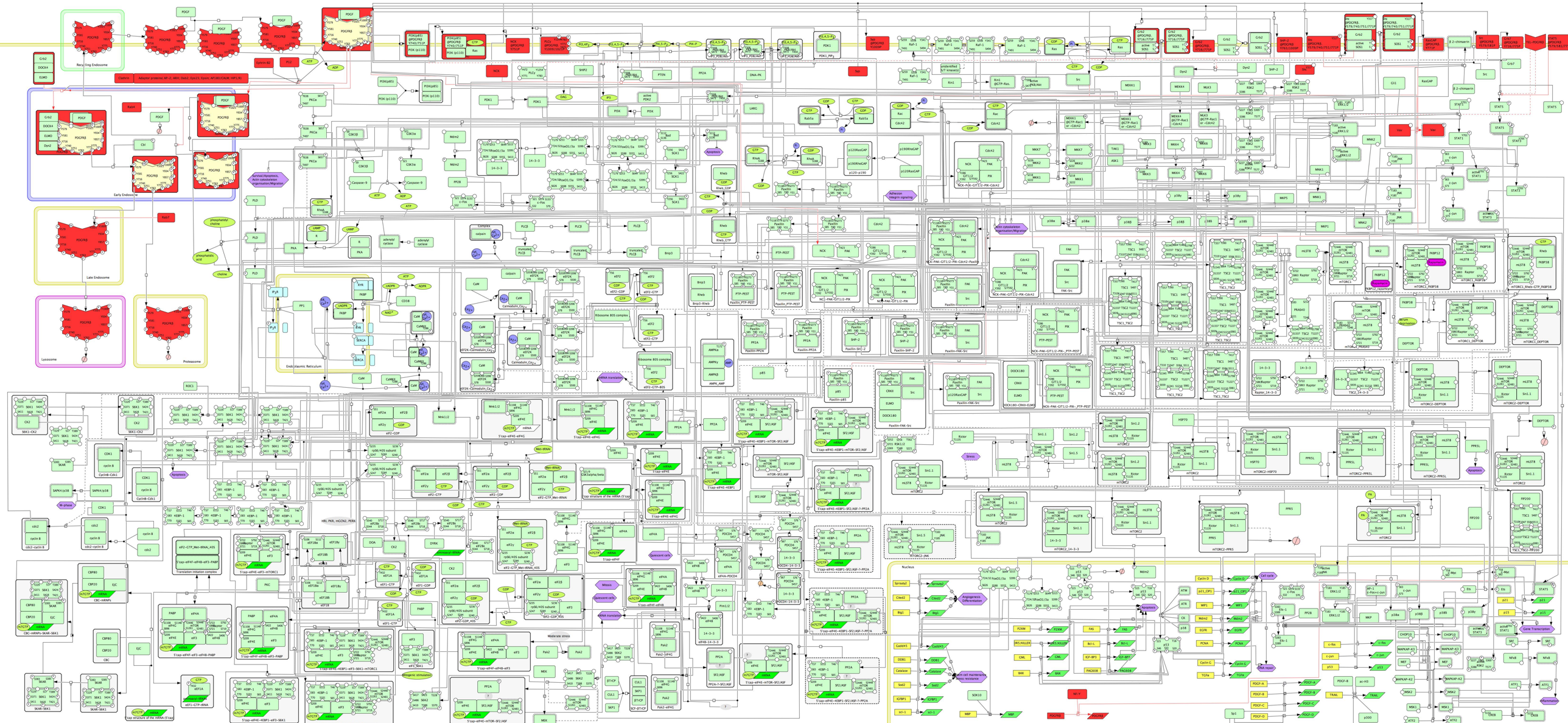

Supplement: Supplementary file 1 [file LSA-2018-00029_Preparation_of_compounds.zip › LSA-2018-00029_Data_file_4_(suppl_material).PDF_format_file_for_PDGFRββ comprehensive_map._Figure_Data_file_4_(suppl_material)..pdf]

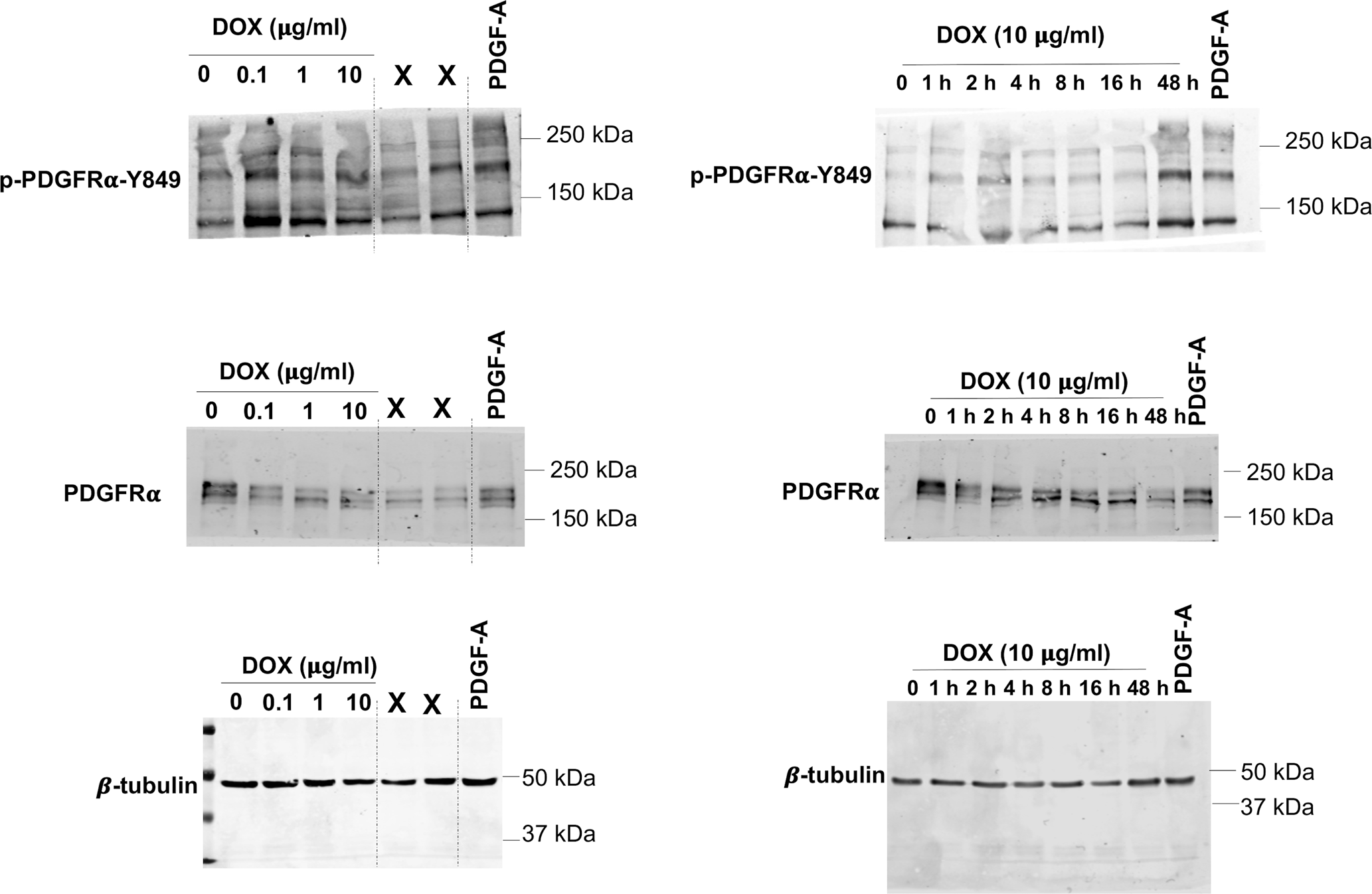

Supplement: Supplementary file 4 [file LSA-2018-00029_SdataF1.tif]

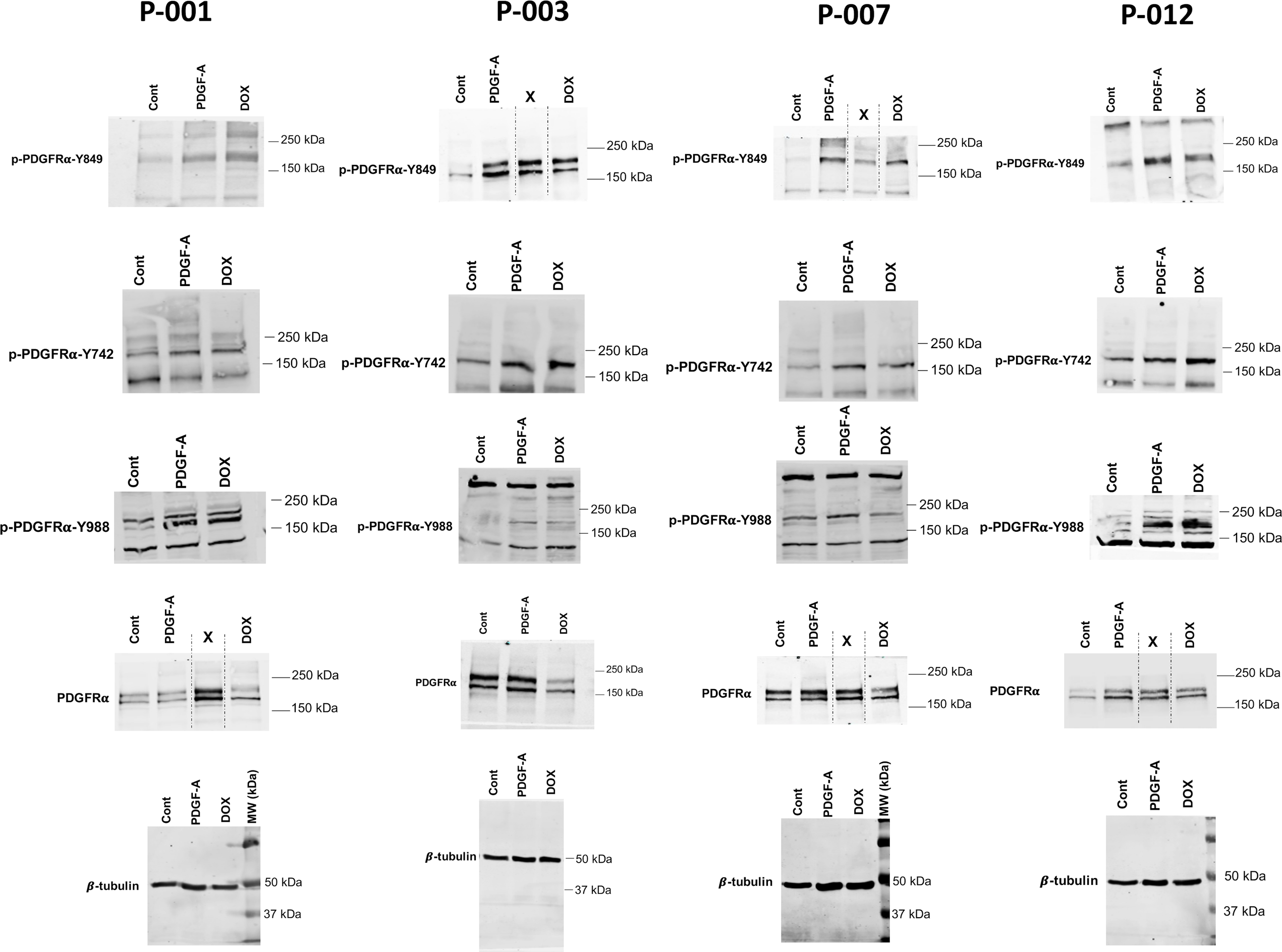

Supplement: Supplementary file 5 [file LSA-2018-00029_SdataF2.tif]

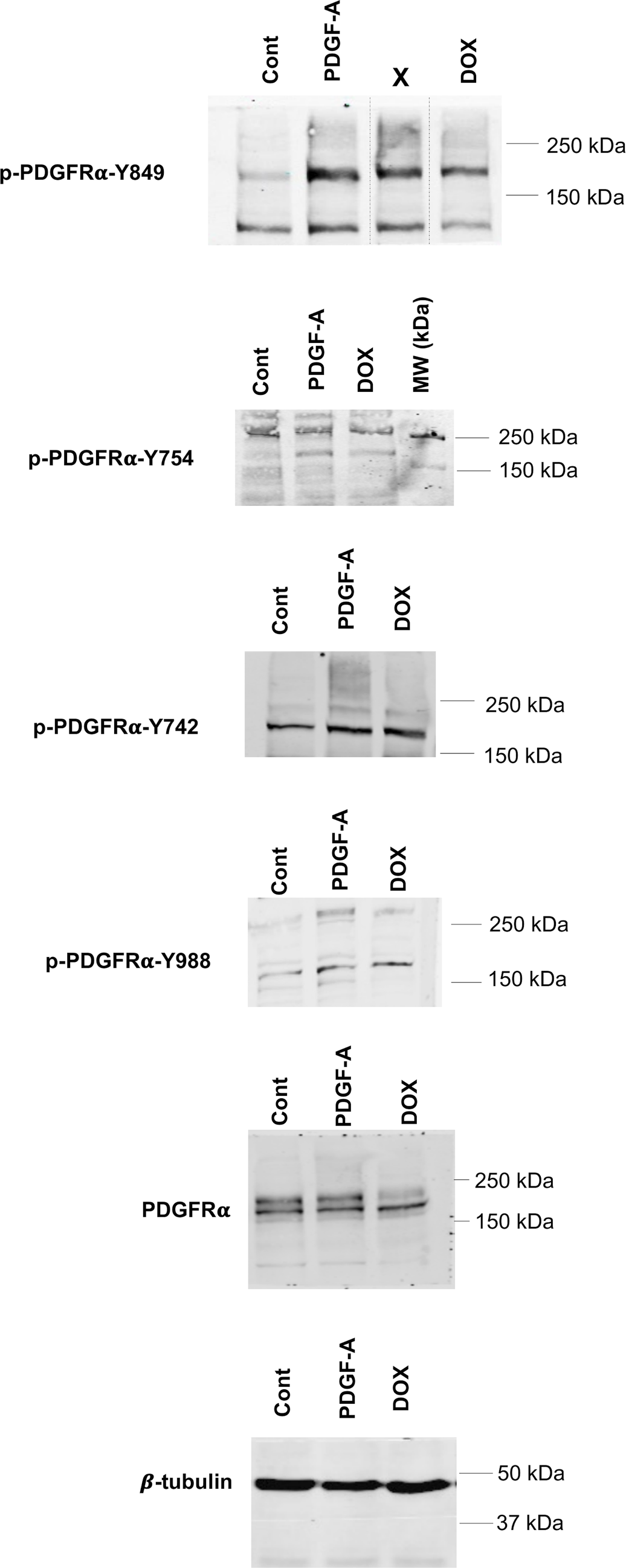

Supplement: Supplementary file 6 [file LSA-2018-00029_SdataF3.tif]

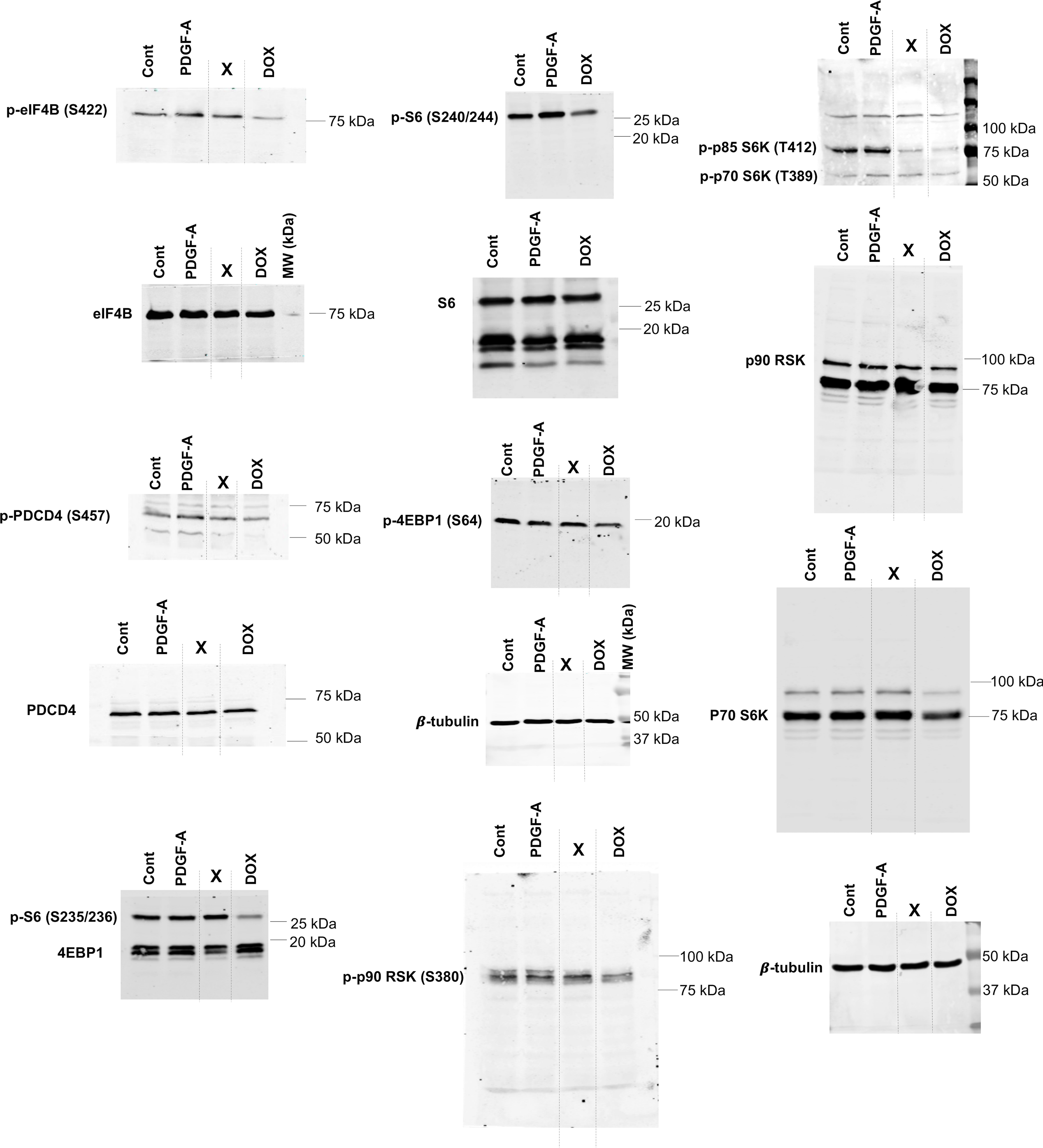

Supplement: Supplementary file 10 [file LSA-2018-00029_SdataF4.tif]

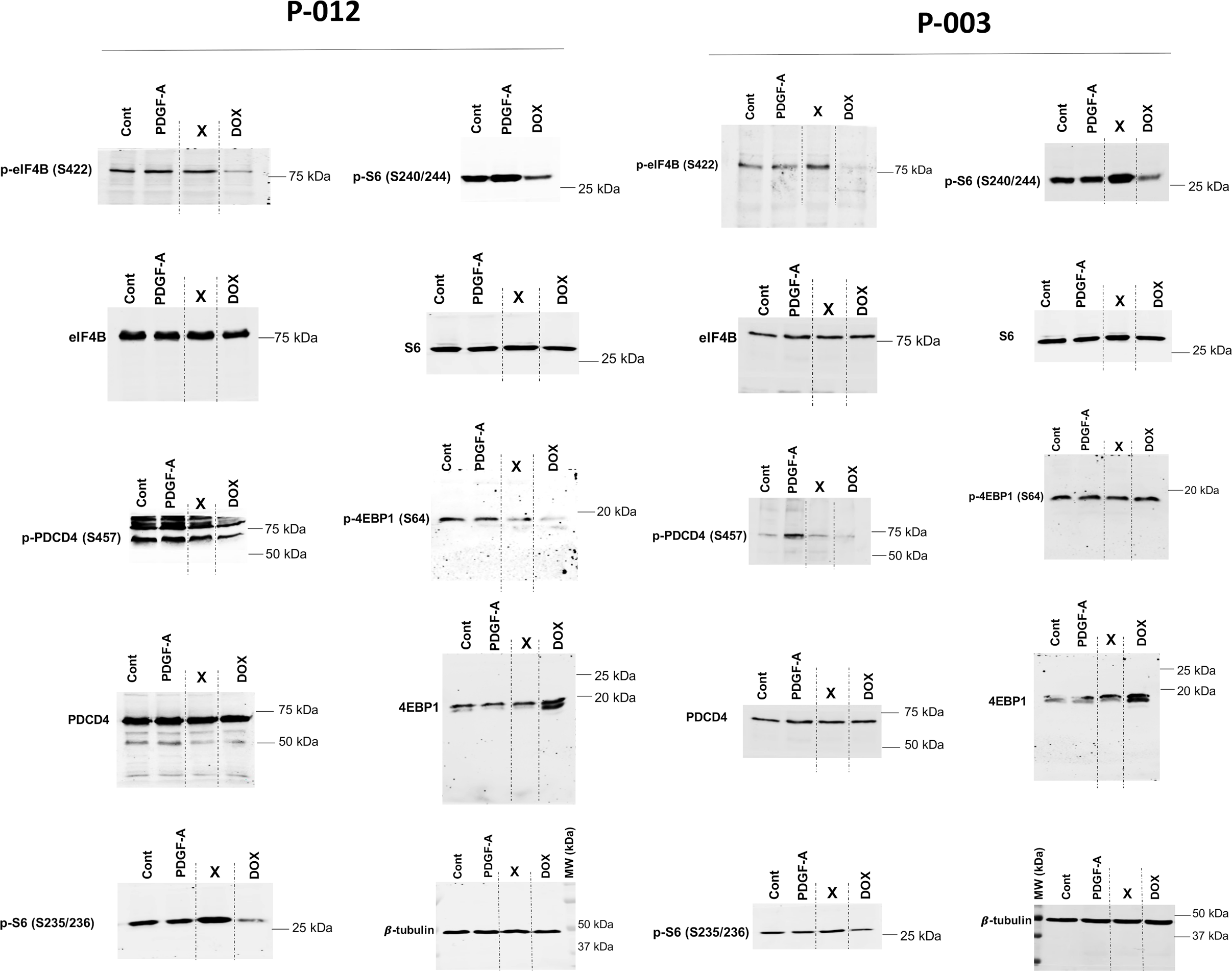

Supplement: Supplementary file 11 [file LSA-2018-00029_SdataF5.tif]
