# Supplementary material for: Chronic platelet-derived growth factor receptor signaling exerts control over initiation of protein translation in glioma
Source: Life Sci Alliance. 2018 Jun 19;1(3):e201800029. doi: 10.26508/lsa.201800029 (PMC6238596; doi:10.26508/lsa.201800029)
Supplement: Supplementary file 2 [file LSA-2018-00029_TableS1.pdf]

Table S1. Elements in PDGFR comprehensive maps.

Species	PDGFRαα	PDGFRββ
Proteins	322	326
Complexes	163	160
Simple molecules	21	21
Genes	37	37
RNAs	39	38
Ions	5	5
Degraded products	9	8
Unknown molecules	3	3
Phenotypes	16	16
**Species in Compartments**	**PDGFRαα**	**PDGFRββ**
Cytoplasm/Plasma membrane	437	452
Nucleus	151	138
Recycling endosome	1	1
Early endosome	8	8
Late endosome	2	1
Lysosome	4	2
Proteasome	2	2
Endoplasmic reticulum	9	9
Extracellular environment	1	1
**Reactions**	**PDGFRαα**	**PDGFRββ**
Heterodimer associations	100	102
State transitions	218	218
Known transitions omitted	82	82
Dissociations	16	16
Transports	25	23
Negative influence	1	1
Triggers	5	5
Unknown transitions	1	1
