# Supplementary material for: Chronic platelet-derived growth factor receptor signaling exerts control over initiation of protein translation in glioma
Source: Life Sci Alliance. 2018 Jun 19;1(3):e201800029. doi: 10.26508/lsa.201800029 (PMC6238596; doi:10.26508/lsa.201800029)
Supplement: Supplementary file 3 [file LSA-2018-00029_TableS2.pdf]

Table S2. Reaction coverage of PDGFRα map in Reactome and Phosphosite Plus

**Reactions**	**Total**	**Reactome**	
		**Covered**	**Not covered**
Heterodimer associations	100	60	40
State transitions	218	145	73
Known transitions omitted	82	82	0
Dissociations	16	9	7
Transports	25	15	10
Negative influence	1	0	1
Triggers	5	0	5
Unknown transitions	1	0	1
**State transitions**	**Total**	**Reactome**	
		**Covered**	**Not covered**
Phosphorylation	153	104	49
Activation	6	4	2
Degradation	7	4	3
Dephosphorylation	20	9	11
Dimerization	4	4	0
Hydrolysis	3	3	0
Nucleotide exchange	15	15	0
Ubiquitination	10	2	8
**Phosphorylation sites**	**Total**	**PhosphoSite Plus**	
		**Covered**	**Not covered**
Phosphorylation sites	189	180	9
