# Supplementary material for: Chronic platelet-derived growth factor receptor signaling exerts control over initiation of protein translation in glioma
Source: Life Sci Alliance. 2018 Jun 19;1(3):e201800029. doi: 10.26508/lsa.201800029 (PMC6238596; doi:10.26508/lsa.201800029)
Supplement: Supplementary file 7 [file LSA-2018-00029_TableS3.pdf]

Table S3. Most differentially regulated phosphorylation sites upon chronic vs. acute stimulation of PDGFRα

Top 10 activated p-sites				
**Gene**	**P-sites**	**DOX**	**PDGF-A**	**Differential**
Ptrf	S302	0.26	-1.64	1.89
Sun2	S281	0.79	-1.08	1.87
Hnrnpa1	369	-0.02	-1.86	1.84
Pcnp	S147	0.19	-1.62	1.81
Pdlim7	S111	0.05	-1.75	1.80
Gigyf2	S140	0.97	-0.80	1.77
Ahnak	S5563;T5567	-0.51	-2.27	1.76
Ahnak	S5553;S5563;T5567	-0.43	-2.12	1.69
Pvr	S384	0.46	-1.18	1.65
Wdr47	S312	0.66	-0.99	1.65
**Top 10 decreased p-sites**				
**Gene**	**P-sites**	**DOX**	**PDGF-A**	**Differential**
Srrm2	S1067	0.00	3.05	-3.05
Cdkn2aip	S124	-0.05	2.48	-2.54
Hmgn1	S7	-0.23	2.28	-2.51
Fbln2	S574	0.16	2.38	-2.22
Rbm15	S700	0.93	3.13	-2.20
Tns1	S1067;S1070	0.44	2.59	-2.15
Iws1	S666	-0.20	1.91	-2.11
Iws1	T668	-0.21	1.88	-2.09
Nab2	S367	-0.50	1.56	-2.06
Nop56	S554	0.12	2.14	-2.03
