# Supplementary material for: Chronic platelet-derived growth factor receptor signaling exerts control over initiation of protein translation in glioma
Source: Life Sci Alliance. 2018 Jun 19;1(3):e201800029. doi: 10.26508/lsa.201800029 (PMC6238596; doi:10.26508/lsa.201800029)
Supplement: Supplementary file 8 [file LSA-2018-00029_TableS4.pdf]

Table S4. Kolmogorov-Smirnov test of Pathway Regulation upon chronic vs. acute stimulation of PDGFRα in different functional groups.

Pathway	D-value	P-value
Cap-dependent translation	0.236111111	0.036125814
Ca2+ signaling	0.333333333	0.099561848
MAPK signaling	0.318181818	0.215373874
PI3K signaling	0.318181818	0.215373874
Cytoskeleton Dynamic	0.161764706	0.335853629
Unknown	0.375	0.627167042
mTORC1	0.5	0.699374199
Small GTPase	0.185185185	0.743544609
Transcription and cell cycle	0.092783505	0.797930976
Other	0.230769231	0.879324397
Endocytosis and recycling	0.25	0.963945244
mTORC2	0.5	0.963945244
STAT & Receptors	0.166666667	0.996255192
