# Supplementary material for: Chronic platelet-derived growth factor receptor signaling exerts control over initiation of protein translation in glioma
Source: Life Sci Alliance. 2018 Jun 19;1(3):e201800029. doi: 10.26508/lsa.201800029 (PMC6238596; doi:10.26508/lsa.201800029)
Supplement: Supplementary file 9 [file LSA-2018-00029_TableS5.pdf]

Table S5. Kolmogorov-Smirnov test of kinase regulation upon chronic vs. acute stimulation of PDGFRα.

**Kinase**	**D-value**	**P-value**
MTOR	*0.429*	*0.003*
P90RSK	*0.692*	*0.004*
ERK2	*0.293*	*0.014*
JNK3	*1.000*	*0.037*
ERK1	0.357	0.056
CDK2	0.314	0.063
CK2A1	0.273	0.076
RET	0.800	0.082
PKCD	0.500	0.100
ABL	0.556	0.124
P70S6K	0.556	0.124
