# Supplementary material for: Chronic platelet-derived growth factor receptor signaling exerts control over initiation of protein translation in glioma
Source: Life Sci Alliance. 2018 Jun 19;1(3):e201800029. doi: 10.26508/lsa.201800029 (PMC6238596; doi:10.26508/lsa.201800029)
Supplement: Supplementary file 12 [file LSA-2018-00029_TableS6.pdf]

Table S6. Information on antibodies used in immunoblot analyses.

Antibody-Targets	Source	Catalog #	Dilution
**β-tubulin**	Sigma	T4026	1 : 5000
**Phospho-PDGFRα (Y849)**	CST	3170	1: 1000
**Phospho-PDGFRα (Y754)**	CST	2992	1:500
**Phospho-PDGFRα (Y742)**	GeneTex	GTX25452	1:500
**Phospho-PDGFRα (Y988)**	Bioss	Bs-5532R	1:500
**PDGFRα**	CST	5241	1: 1000
**Phospho-eIF4B (S422)**	CST	3591	1: 1000
**eIF4B**	CST	3592	1: 1000
**Phospho-PDCD4 (S457)**	ROCKLAND-INC	600-401-964	1: 1000
**PDCD4**	CST	9535	1: 1000
**Phospho-S6 (S240/244)**	CST	2215	1: 1000
**Phospho-S6 (S235/236)**	CST	2211	1: 1000
**S6**	CST	2217	1: 1000
**Phospho-4EBP1 (S64)**	CST	9451	1: 1000
**4EBP1**	CST	9452	1: 1000
**Phospho-p90 RSK (S380)**	CST	9341	1: 1000
**p90 RSK**	CST	9355	1: 1000
**Phospho-p85 RSK (T412)**	CST	9206	1: 1000
**Phospho-p70 RSK (T389)**	CST	9206	1: 1000
**p70 S6K**	CST	9202	1: 1000
